# Nano-antimicrobial peptides (Nano-AMPs) to combat resistant gram-negative bacteria

**DOI:** 10.1007/s13346-026-02085-x

**Published:** 2026-03-02

**Authors:** Naveed Saleem, Naresh Kumar, Emad El-Omar, Mark Willcox, Xiao-Tao Jiang

**Affiliations:** 1 Microbiome Research Centre, School of Clinical Medicine, St George and Sutherland Clinical Campuses, University of New South Wales, (UNSW), Sydney, NSW Australia; 2 School of Chemistry, University of New South Wales (UNSW), Sydney, NSW Australia; 3 School of Optometry and Vision Science, The University of New South Wales, Sydney, NSW Australia

**Keywords:** Antimicrobial peptides (AMPs), Antimicrobial resistance (AMR), Drug-delivery systems, Gram-negative pathogens, Nanocarriers

## Abstract

The emergence of antimicrobial resistance (AMR) poses a critical threat to public health worldwide, making conventional antibiotics ineffective against multidrug-resistant (MDR) pathogens. This literature review examines the potential therapeutic applications of nano-antimicrobial peptides (Nano-AMPs), with a focus on multidrug-resistant pathogens prioritized by the World Health Organisation (WHO). Antimicrobial peptides (AMPs) are essential components of the innate immune system with broad-spectrum bactericidal and immunomodulatory properties, and have emerged as promising alternatives to conventional antibiotics because of their unique mechanisms of action (e.g., membrane disruption, pore formation, and immunomodulation). Currently, the clinical translation of AMPs is hindered by several challenges, including enzymatic and non-enzymatic degradation, poor bioavailability, and biocompatibility issues, as well as local and systemic adverse events. To address these concerns, recent advancements in nanocarrier delivery systems offer novel solutions, enabling selected and targeted drug delivery, enhanced bioavailability, and controlled and sustained AMP release. Lipid-based nanocarriers (e.g., liposomes), polymeric and other nanocarrier systems improve peptide solubility and limit off-target events, while inorganic carriers like gold, silver, and silica nanoparticles facilitate functionalization and synergism to combat MDR Gram-negative infections. Despite promising findings, challenges such as production, long-term efficacy and safety, and regulatory approval persist. Therefore, interdisciplinary efforts, such as advanced machine learning methods alongside conventional pharmacological approaches, may be needed to optimize nanocarrier designs and validate clinical efficacy and safety in preclinical and clinical trials. This review critically analyses the latest evidence on different nanocarriers and their synergistic effects, highlighting their transformative potential to combat AMR, thereby offering insights to develop next-generation antibiotics, particularly against Gram-negative pathogens.

## Introduction

**Fig. 1:**
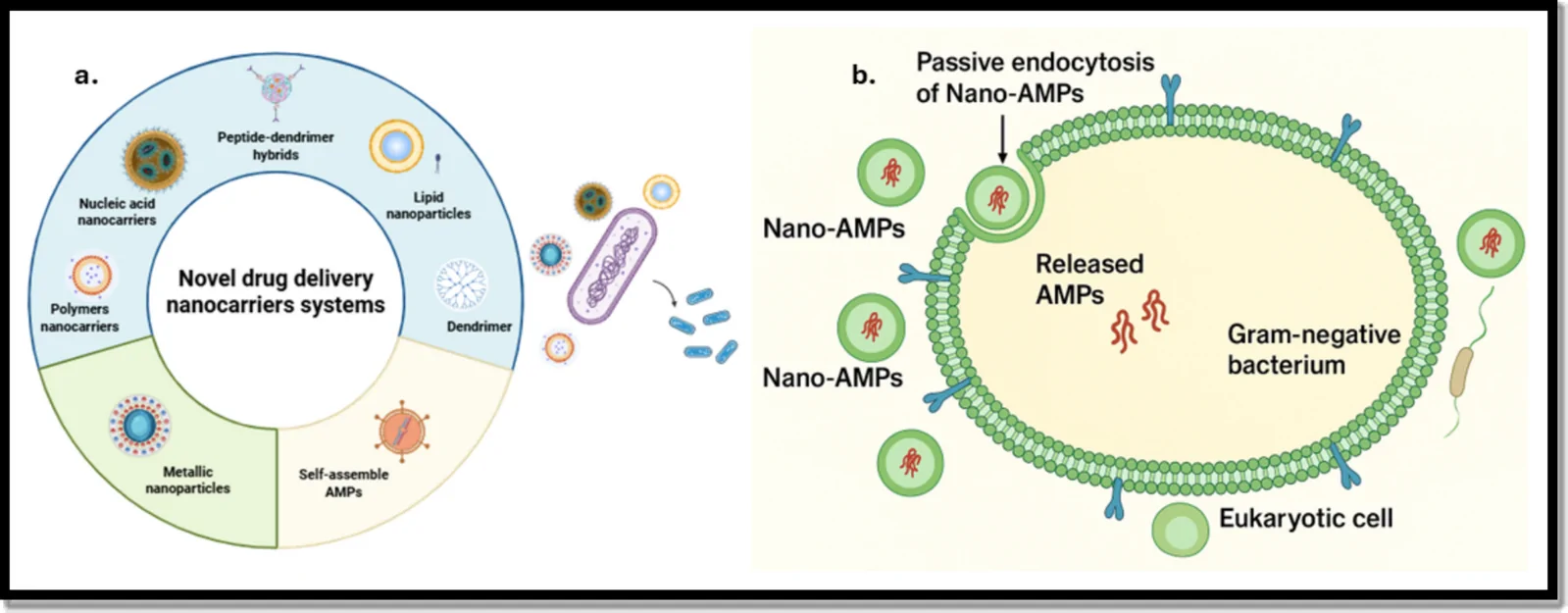
Graphical abstract demonstrating a schematic representation of different nano-drug delivery approaches. **a** Nano-carriers such as polymers, liposomes, cyclodextrins, nucleic acids, dendrimers, self-assemble, and metallic hybrids. **b** These drug delivery approaches can be engineered to enhance AMP targeting, membrane penetration, and stability. The passive targeting route enables Nano-AMPs to accumulate within the infected tissues and interact with microbial membranes, improving delivery and antimicrobial potency. The image was created with BioRender. (https://www.biorender.com)

Antimicrobial resistance (AMR), particularly against Gram-negative pathogens, poses a serious global health threat, thereby impacting effective treatment against infectious diseases [1, 2]. WHO has declared AMR a top global health priority because of rising AMR prevalence and severe clinical and economic consequences [2–4], and identified several antibiotic-resistant Gram-negative bacteria as priority targets for the development of new therapies, underscoring the urgent need for innovative solutions [3, 4]. By 2050, AMR is projected to cause 10 million deaths annually, with Gram-negative bacteria being among the critical contributors to this crisis [3, 5]. The emergence of multidrug-resistant (MDR) and extensively drug-resistant (XDR) strains has led to a scarcity of available treatment options, resulting in increased morbidity and mortality, prolonged hospital stays, and higher healthcare costs [1–3]. Despite efforts to develop new antibiotics, the pipeline is still limited, necessitating alternative therapeutic strategies [1–3] (Fig. 1).

AMPs are either natural or synthetic (usually cationic and amphipathic) molecules that can selectively bind to anionic bacterial membranes, causing pore formation, membrane permeabilization, and microbial lysis [6–15]. Certain AMPs penetrate bacterial membranes and target intracellular components such as proteins, RNA, or DNA, thereby disrupting key cellular functions [7–10]. For example, LL-37 and indolicidin are known to bind bacterial DNA [16–20]. Once intracellular, AMPs can inhibit enzymes crucial for bacterial metabolism, thereby causing cellular dysfunction [16–20].

**Fig. 2:**
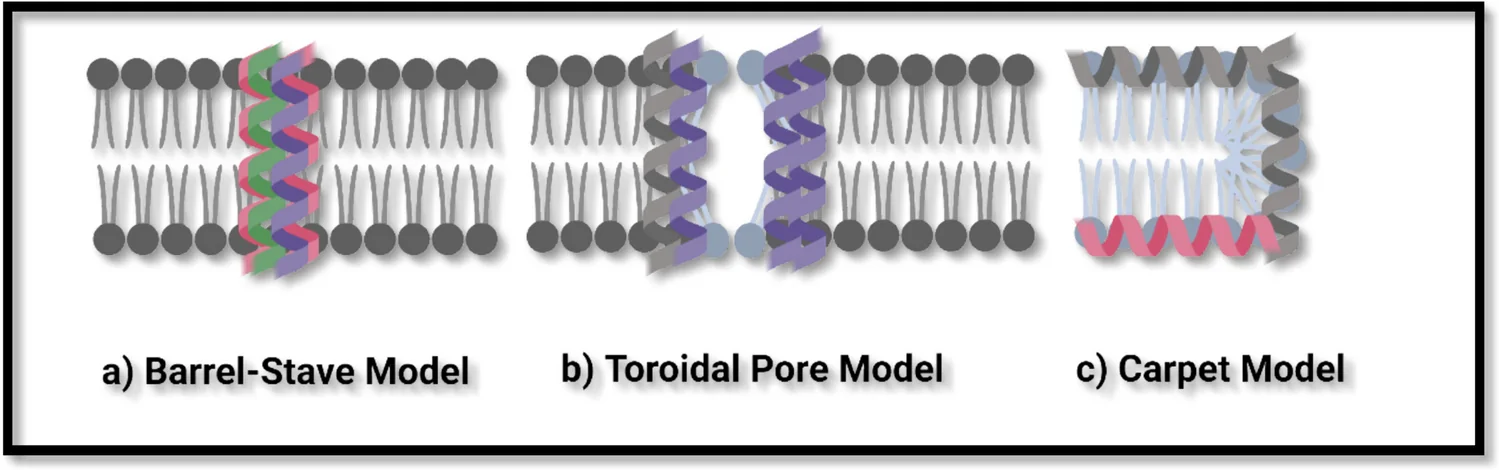
Models of action for AMPs. **a** Barrel-stave model: AMPs aggregate into multimers to form transmembrane channels inserted within the lipid bilayer and align parallel to the phospholipids. **b** Toroidal pore Model: AMPs embed perpendicularly within the membrane, bending to form a continuous porous structure through the lipid bilayer. **c** Carpet model: AMPs accumulate on the microbial membrane surface, acting similarly to detergents, leading to bacterial membrane disruption and cell lysis. Image was created with BioRender. (https://www.biorender.com)

Anionic lipopolysaccharides (LPS) present at the outer membrane of Gram-negative bacteria attract cationic AMPs, which can disrupt the membrane by inserting within the membrane to form transmembrane pores (**Barrel-Stave Model; **Fig. 2a), or they can also create pores by bending the membrane around themselves (**Toroidal Pore Model; **Fig. 2b) [21]. Moreover, this process can disrupt membrane integrity by covering the membrane like a "carpet" through micelle formation (**Carpet Model; **Fig. 2c) [21]. These disruptive approaches can cause the loss of membrane potential or leakage of intracellular contents, eventually leading to bacterial cell death [21].

Many AMPs are present as inactive precursors (called ‘’pro-AMPs’’) and often require enzymatic processing, such as with proteases, for their activation. AMPs function as a first-line defence against invading pathogens before the adaptive immune system activation [19, 20]. Bacterial infections can induce changes within *Proteasome* composition and function, including *Proteasome activator subunit 3* (PSME3) recruitment, increasing cleavage, and thereby enhancing bactericidal activity [19, 20]. Despite promising therapeutic advantages, certain challenges, such as higher cost of production, regulatory hurdles, poor bioavailability and cytotoxic adverse events, must be addressed to enable clinical translation [6–10]. Some of these may be overcome by strategic efforts to optimize AMP delivery systems, including nanocarriers to produce Nano-AMPs.

Machine learning (ML) and artificial intelligence (AI) can facilitate peptide design and synthesis with improved specificity, stability, and potency [22, 23]. Generative AI frameworks can be extended to optimize AMP sequences for carrier biocompatibility and immune stealth [22, 23]. Altogether, machine learning approaches can be used to predict optimal nanocarrier types and drug loading parameters by correlating their physicochemical properties with biological performance, tailoring nanocarriers to enhance AMP delivery, and overcoming biological barriers [22, 23]. Nano-AMPs could emerge as an effective and sustainable drug delivery solution to the growing global AMR crisis, offering better hopes for the treatment of infectious diseases in the future [12].

This review primarily focuses on the latest in-vitro and in-vivo therapeutic evidence for different organic and inorganic Nano-AMPs, particularly against Gram-negative bacteria. It proposes the integration of the emerging paradigm of AI-driven approaches for novel AMP design alongside nanocarrier engineering (Fig. 1). Nanocarriers such as liposomes, polymeric, and metallic nanoparticles are being developed to enhance AMP stability, selective and targeted delivery, and improving therapeutic efficacy [12] (Fig. 1). Nano-AMPs also show synergistic effects along with pre-existing antibiotics, thereby potentially restoring their clinical efficacy against resistant and life-threatening Gram-negative bacteria [12]. Combination therapies may also minimize antimicrobial resistance development [24].

The current review provides a focused examination of Nano-AMPs as an innovative solution to AMR delivery, and highlights the need to explore advanced multi-model strategies for machine learning-guided AMPs and nanocarrier designs. This paper also proposes novel drug delivery strategies by leveraging next-generation AI tools to predict both AMP scaffolds and their nanocarrier shells. Additionally, it presents forward-looking perspectives by linking innovative AMP sequential designs, strategic nanocarrier selection and synthesis, and translational potential, framed within the context of current clinical resistance trends.

### Gram-negative dual-membrane architecture creates barriers for conventional drugs

**Fig. 3:**
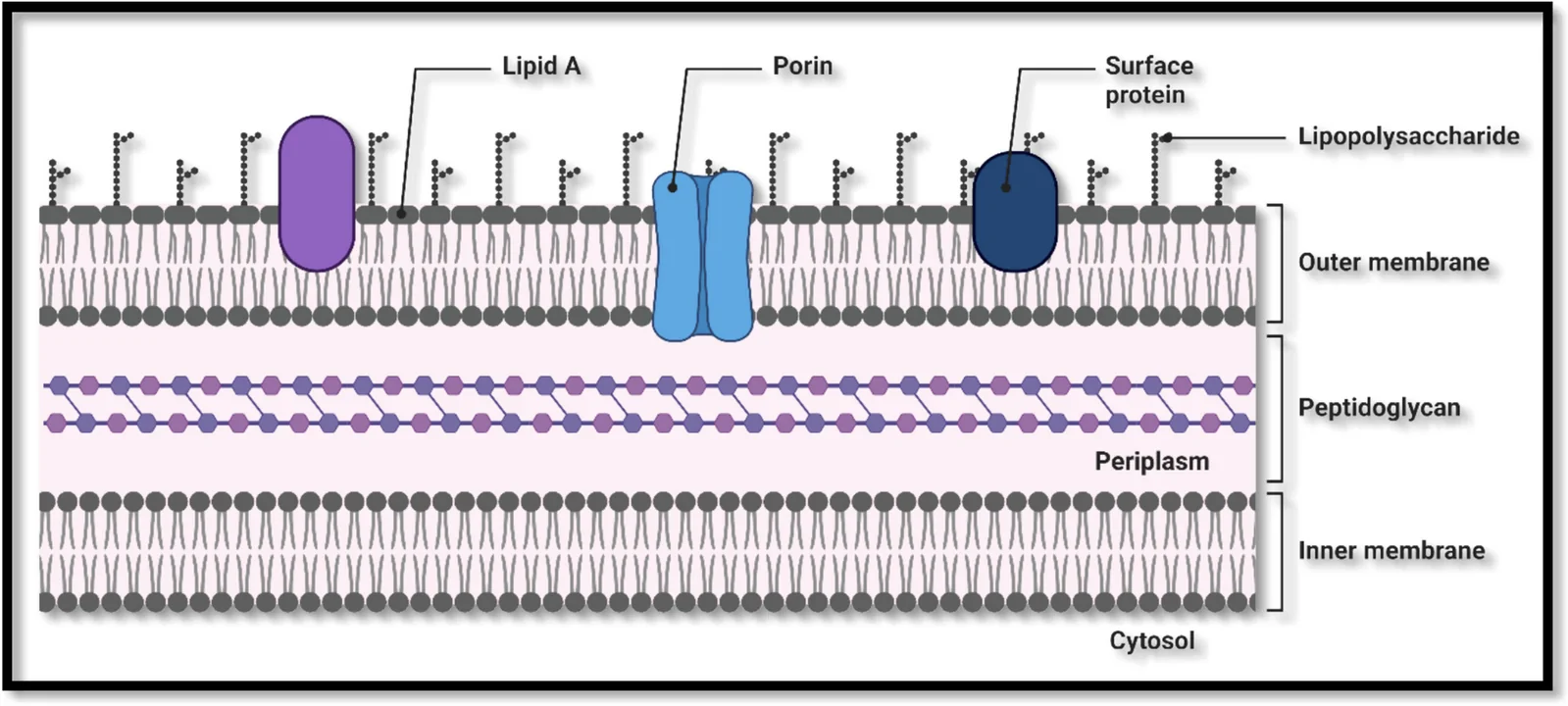
Structure of Gram-negative bacterial membrane. Gram-negative bacteria pose greater clinical challenges due to a unique double-membrane architecture. The inner cytoplasmic membrane is composed primarily of phospholipids and integral transport proteins, whereas the outer membrane (OM) possesses an asymmetric bilayer with lipopolysaccharide (LPS) at its outer leaflet, acting as a permeability barrier to larger biomolecules and hydrophobic drugs. Porins are embedded within the outer membrane, further restricting AMP and antibiotic entry depending on charge-selective channels. A thin peptidoglycan cell wall, sandwiched in the periplasmic space, lies beneath these layers and contributes to sequestering β-lactam-targeted enzymes and osmotic stability. Created with BioRender. (https://www.biorender.com)

Emergence of multidrug-resistant strains of Gram-negative bacteria, such as *Acinetobacter baumannii*, *Escherichia coli, Pseudomonas aeruginosa*, and *Klebsiella pneumoniae,* has exacerbated the global antimicrobial resistance (AMR) crisis [3–5]. These pathogens are often resistant due to their complex cell structures, reduced membrane permeability, degradation of antibiotics, presence of efflux pumps and porins, and ability to acquire resistance genes through horizontal gene transfer (HGT) [25–27] (Fig. 3).

**Fig. 4:**
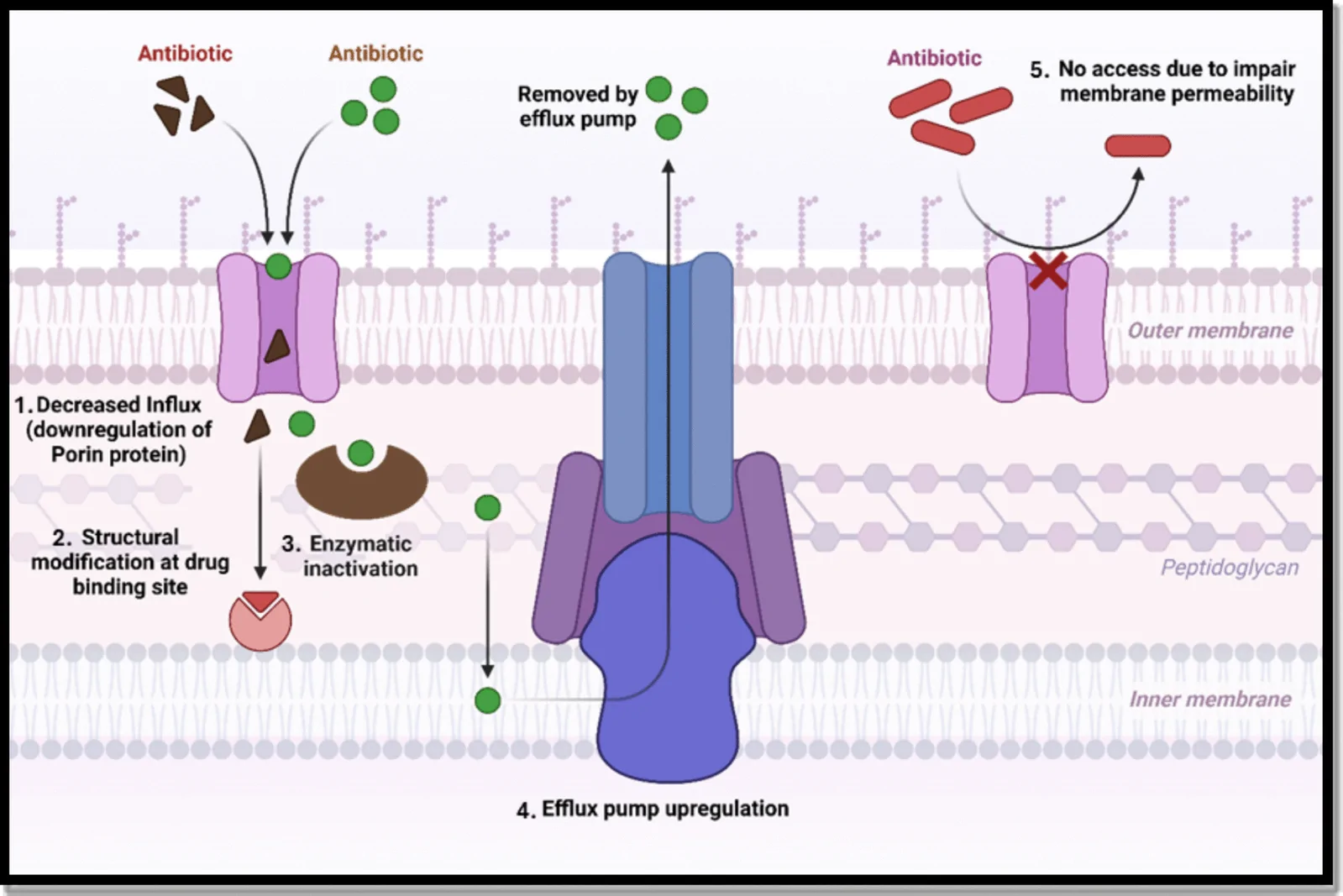
General mechanisms of acquired resistance. Gram-negative bacteria adopt the following mechanisms of acquired resistance through synergistic mechanisms that can reduce drug uptake and enhance clearance, against conventional antibiotics: 1) downregulation of porin proteins limits permeability, 2) structural alteration of drug receptors decreasing drug binding affinity, 3) upregulation of inactivating enzymes such as β-lactamases which neutralize antibiotic activity, 4) efflux pump upregulation, expel drugs from the bacterial cell, favouring resistance across Gram-negative pathogens, and 5) decreased antibiotic influx due to membrane permeability due to alterations in lipid composition. These mechanistic approaches act synergistically to maintain intracellular antibiotic concentrations below therapeutic levels, underpinning multidrug resistance trends amongst Gram-negative pathogens. The image was created with BioRender. (https://www.biorender.com)

The glycans on lipopolysaccharide (LPS) molecules present in the outer leaflet of the outer membrane can function as a barrier to the interactions of cationic AMPs with underlying anionic lipids and the ability of antibiotics to penetrate cells (Fig. 3) [25, 28]. Porins are protein channels embedded within the outer membrane and regulate the entry of small molecules, including antibiotics (Fig. 4) [25]. Certain antibiotics are hydrophobic or too large to pass through porins, limiting their access to the bacterial periplasm and cytoplasm [27]. Bacteria can either alter porin structure or downregulate porin expression to limit antibiotic uptake [25, 27].

Intrinsic resistance genes in Gram-negative bacteria can encode proteins that make up efflux pumps or enzymes that degrade antibiotics. Efflux pumps actively expel antibiotics and other toxic substances out of the bacterial cells, thereby reducing intracellular drug concentrations and rendering antibiotics ineffective (Fig. 4) [25, 26]. Multidrug efflux pumps such as AcrAB-TolC in *E. coli* can remove multiple classes of antibiotics, contributing to multidrug resistance [26].

Gram-negative bacteria can produce enzymes that can either modify or degrade antibiotics, such as β-lactamases that hydrolyze β-lactam antibiotics (penicillin, carbapenems, and cephalosporins), and aminoglycoside-modifying enzymes that inactivate aminoglycosides by acetylation, phosphorylation, or adenylation (Fig. 4) [25, 29]. Gram-negative bacteria can also readily exchange genetic material through processes including conjugation, transformation, and transduction, facilitating the rapid acquisition of resistance genes, such as carbapenemases or those encoding extended-spectrum β-lactamases (ESBLs) [25, 30]. Overcoming these challenges requires innovative strategies, such as Nano-AMPs, to target them effectively and circumvent their resistance mechanisms.

### Nanocarriers as alternative drug delivery systems

AMPs are part of the innate immune system, usually formed of 10–100 amino acids, and are characterized by an amphipathic nature and cationic charge, allowing them to interact with the anionic microbial membranes [7–10]. Whilst AMPs have many important properties, such as the difficulty of bacteria becoming resistant to them [14]. Their clinical applications are hampered as they are prone to enzymatic degradation by proteases in-vivo [31], serum binding and rapid systemic clearance [15]. Higher concentrations of AMPs may cause host cytotoxicity, especially in sensitive tissues [14]. Moreover, the chemical synthesis of AMPs at a larger scale is expensive, posing a barrier to commercialization [15].

**Fig. 5:**
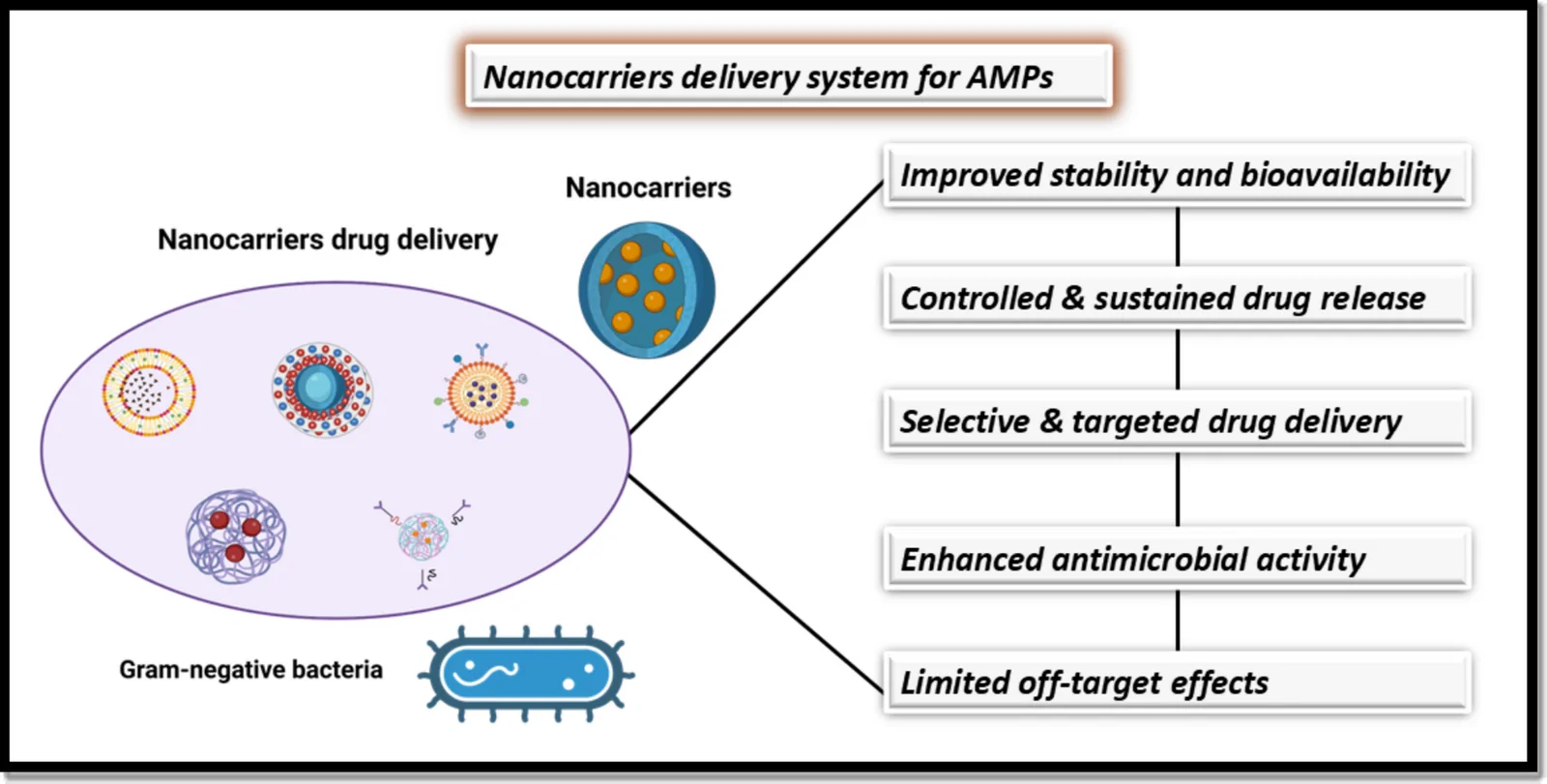
Incorporation of AMPs within different drug delivery nanocarriers can enhance the physicochemical stability, bioavailability, and therapeutic index of AMPs. Nanocarrier encapsulation facilitates selective and targeted delivery to infection sites, enhancing their antimicrobial efficacy, minimising host off-target and adverse effects, and supporting safer systemic administration routes. Image was created with BioRender. (https://www.biorender.com)

Incorporation of AMPs within the nanocarriers is critical for addressing the limitations associated with free AMPs, such as poor pharmacokinetic and pharmacodynamic profiles, particularly in the context of multidrug-resistant Gram-negative pathogens (Fig. 5) [32–35]. Nanocarriers may be able to protect AMPs from enzymatic and non-enzymatic degradation, facilitating their interactions specifically with microbial cells, thereby increasing antimicrobial activity and limiting off-target effects (Fig. 5) [32–35]. Moreover, they may possess immunomodulating properties, facilitating the recruitment of immune cells and favouring cytokine production, thereby enhancing their therapeutic potential [7–10].

#### Improved bioavailability at a site of infection

Nanocarriers can improve the bioavailability of AMPs at infection’s sites by addressing key pharmacological challenges, by facilitating controlled AMP release, maintaining therapeutic concentrations over an extended period, thereby enhancing circulation half-life [31]. Nano-AMPs can favour the absorption and retention of AMPs at the infection site, thereby reducing dosage frequency [31, 35].

Nanosystems, such as solid lipid nanoparticles (SLNs), lipid and polymeric nanoparticles, can be used to avoid enzymatic degradation by encapsulating AMPs within a biocompatible matrix, thereby prolonging their systemic circulation and enabling sustained release at the infection site [31]. For instance, encapsulating colistin in lipid nanoparticles can reduce nephrotoxic events and increase plasma half-life from 2 to 8 h while enhancing lung accumulation by threefold in *P. aeruginosa* infections [36, 37].

Lipid nanoparticles encapsulating polymyxin B showed sustained drug levels and higher pulmonary tissue concentrations in an infected rat lung infection model, with significant bacterial clearance of *P. aeruginosa,* as well as protecting the drug from rapid systemic elimination, which concentrated the AMPs at the infection site [38].

In a murine pneumonia model, encapsulation of polymyxin B within lipid nanoparticles increased drug retention and exposure timings within the pulmonary epithelial linings, enabling efficient microbial killing at smaller doses, establishing lipid-based nanocarriers as a powerful strategy for enhancing local AMP bioavailability, particularly in pulmonary infections [39].

#### Selected and targeted delivery

Nanocarriers can deliver AMPs across the outer membranes of Gram-negative bacteria, bypassing challenges posed by LPS [14, 15]. Functionalized nanoparticles can specifically interact with microbial membrane components (e.g., LPS-binding motifs, porins), enhancing cellular binding and uptake [14, 15]. They can be optimized using targeting ligands such as antibodies and recognizable bacterial-specific receptors, enabling precision delivery [40]. Peptide amphiphiles underwent self-assembly when functionalized with a heparin-binding cardin-motif peptide (sequence (AKKARK)2) to combat drug-resistant pathogens by disorganizing their outer membranes, causing cytoplasmic leakage [41].

Superparamagnetic iron oxide engineered nanoparticles coated with chitosan formed bioconjugates, enabled covalent immobilization of AMPs, preserved their structural integrity and displayed enhanced bactericidal activity against pathogenic *E. coli* (O157:H7), indicating potential peptide–pathogen interactions [42]. Moreover, the SPION cores exhibited superparamagnetic behaviour due to magnetite structure, supporting external manipulations, highlighting their potential for magnetically guided targeted drug delivery at the infection site [42].

Nano-AMPs functionalized with cadmium selenide (CdSe) displayed better antimicrobial activity, killing > 98% bacterial population in-vitro against multidrug-resistant *E. coli* and significant microbial clearance in-vivo, because of the enhanced peptide’s water solubility, favouring stability, with stronger membrane disruptive properties [43]. Moreover, haematological and histopathological analysis showed that the quantum-dot nanocarrier minimized systemic toxicity by reducing off-target effects [43]. Therefore, Nano-AMP can be engineered to deliver highly efficacious and lower-toxicity antimicrobial agents against Gram-negative pathogens [43].

#### Enhanced efficacy and safety

Metallic nanoparticles show intrinsic antimicrobial properties as they can generate ROS, disrupt microbial membranes, interfere with protein function, and impair DNA replication [35]. Incorporation of AMPs within metallic nanocarriers can amplify bactericidal effects and favour synergistic interactions [35]. Metallic nanoparticles functionalize with ligands can improve their cellular uptake via endocytosis, allowing them to reach intracellular pathogens [35]. Stimuli-responsive nanocarriers ensure AMPs are active only at infection sites, such as pH-sensitive AMP conjugates that release melittin within an acidic environment, preventing systemic cytotoxic adverse events [44].

Gold nanoparticle-DNA aptamers favoured intraperitoneal delivery of AMP conjugates (AuNP-Apt-LysAB2 P3-His), with better survival outcomes and significantly lower bacterial loads across major organs amongst mice infected with multidrug-resistant *A. baumannii* [45]. The targeted nanocarrier drug delivery outperformed aptamer-nanoparticles alone or free AMPs by enhancing both pathogen-specific accumulation and peptide stability, demonstrating aptamer-guided AMP nanocarriers can achieve better therapeutic efficacy and safety against untreated Gram-negative infections, underscoring the translational precision of Nano-AMPs for MDR pathogens in-vivo [45].

Silver nanoparticles tagged with cysteine residual AMPs formed bioconjugates, and enhanced efficacy against multidrug-resistant pathogens, particularly against *K. pneumoniae* with MICs 3–tenfold lower than free peptides [46]. Mechanistic approaches and molecular dynamics revealed that nanoparticle conjugates optimized microbial membrane engagement and pore formation are mediated by hydrophobic collapsed methods [46]. Cysteine-guided assembly mitigated the toxicity risks of biocompatible nanostructures by reducing their therapeutic doses, highlighting that Nano-AMPs can achieve higher bactericidal effects with enhanced safety, and provide novel alternatives to drug delivery approaches as compared to conventional antibiotics [46].

#### Limited host cytotoxicity

Nano-AMPs can prevent non-specific interactions with host cell membranes, reducing host cytotoxicity [47]. Functionalized nanocarriers, particularly polymer-based nanosystems incorporating DNA, chitosan, cellulose, or poly (lactic-co-glycolic acid), PLGA, are biocompatible and biodegradable; therefore, they can be used to deliver AMPs specifically to bacterial cells, minimizing non-specific exposure to host cells, and increasing their therapeutic index [35]. PLGA nanoparticles protect AMPs from enzymatic degradation, reducing cytotoxicity by limiting non-specific interactions with non-target cells, thereby optimizing AMP translocation to specific tissues, making them effective carriers for pulmonary infections caused by *P. aeruginosa* [48].

Gold nanorods (AuNRs) functionalized with the AMPs (C-At5) demonstrated better potency while limiting cytotoxicity toward RBCs and human fibroblasts, yielding a more favourable therapeutic window than unconjugated AuNRs [49]. Nanorods-AMP conjugates achieved laser-augmented bactericidal effects at suboptimal nanoparticle therapeutic doses, minimizing non-specific tissue interactions [49]. in-vivo, they demonstrated the biocompatibility of Nanorods-AMPs by accelerating wound healing, highlighting that surface functionalization can be used to convert photothermal cytotoxic agents into relatively safer and efficacious antimicrobial agents [49].

AMPs can modulate immune reactions; therefore, nanoencapsulation can reduce the overall risk of immunogenic responses by shielding the AMPs from immune cells [50]. For instance, gold nanoparticles conjugated with melittin demonstrated up to 90% reduction in *E. coli* loads in a murine sepsis model, attributed to their dual mechanism of membrane disruption and immunomodulation [44]. Polymeric nanoparticles incorporating AMPs have shown promising findings in systemic infection models, where sustained drug release has improved pharmacokinetic profiles, resulting in enhanced survival outcomes while modulating host inflammatory responses [51].

### Strategies to enhance Nano-AMPs' selective targeting

**Fig. 6:**
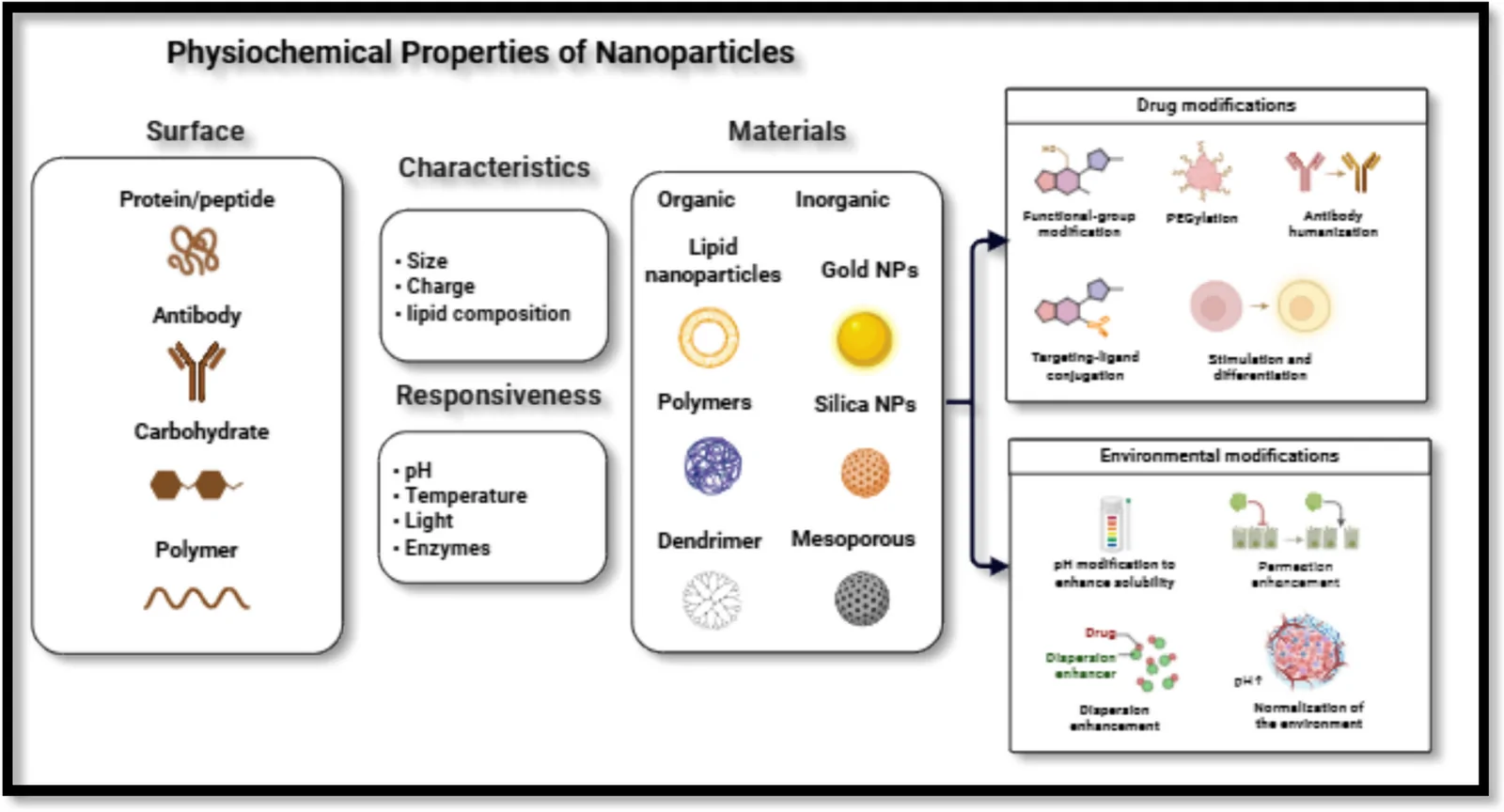
Strategies to enhance Nano-AMPs’ selective and targeted drug delivery approaches, including surface engineering, such as size, charge, and stealth, and ligands for selected bacterial binding. The physicochemical and structural modifications enable membrane interaction. Optimization of AMP, based on environmental factors, such as pH, ROS, enzyme, and light-responsive, facilitates on-site activation and reduces off-target effects. Created in https://BioRender.com

Selective delivery of Nano-AMPs can be of benefit for combating multidrug-resistant infections. At the infection site, there is enhanced vascular permeability [52], enzyme activity, pH, oxidative stress, and immune landscapes, which provide opportunities for targeted drug delivery (Fig. 6). Active targeting strategies include ligands such as mannose, aptamers, folic acid, and antibodies that can bind receptors present either on pathogens or immune cells (e.g., macrophages), significantly enhancing cellular uptake within infected tissues (Fig. 6) (Table 1) [52, 53]. Passive targeting approaches can exploit the enhanced permeability and leakage of infected tissues, favouring extravasation and localization of Nano-AMPs at infection sites. Surface modification and cationicity may enhance penetration through negatively charged cellular matrices (Fig. 6) (Table 1) [52].

**Table 1:** Different targeting strategies to enhance Nano-AMPs' delivery within the infected tissues

Strategy	Mechanism	Advantages
Passive targeting [52]	Smaller-sized nanoparticles, i.e., 100–200 nm, can accumulate within the infected tissues, due to enhanced permeability. Inflamed and leaky vascular and impaired lymphatics favour Nano-AMPs to extravasate and retain locally	Simple targeting route which does not require ligands
Active targeting [53]	Surface ligands bind either to bacterial and host receptors, such as aptamers, folate, mannose, and antibodies	Highly selective and specific, thereby enhancing cellular uptake
Stimuli-responsive release [54]	Triggered by micro-environment stimuli, at the infection site, i.e., enzymes, acidic pH, and ROS	On-demand release, thereby reducing systemic toxicity
Physical targeting [55–58]	Either photo or magnetically responsive nanocarriers deliver to the infection site	There is localized activation, which controls drug release
Biomimetic approaches [59–61]	Either cell membrane–coated or exosome-mediated nanoparticles delivery	It can evade immune systems, innate tissue tropism

Beyond active and passive targeting approaches, stimuli-responsive nanocarriers have emerged as innovative drug delivery systems, releasing AMPs within the diseased microenvironments (Fig. 6) (Table 1). At the infection site, factors such as acidic pH, proteases (either host or bacterial), and redox gradients can trigger controlled and sustained AMPs release from engineered liposomes, polymers, or dendrimers [54].

Physical targeting methods, including magnetically guided nanoparticles [55–58], can also improve the drug’s kinetics. Biomimetic nanocarriers coated with either exosomes or immune-cell membranes may evade immune clearance and reside longer within infected tissues (Table 1) [59–61]. Combining stimulus-responsive modifications alongside host and pathogen-specific ligands can maximize therapeutic potential while minimizing off-target effects.

Furthermore, integration of machine learning and artificial intelligence platforms for AMP discovery and designs can facilitate the co-optimization of AMP sequences and nanocarrier responsiveness, thereby accelerating clinical translation of potentially viable Nano-AMPs against resistant Gram-negative pathogens [62–65].

### Nano-AMPs as novel therapeutics against Gram-negative pathogens

Nanocarriers can significantly improve the efficacy of AMPs by targeting bacterial membranes. Their surface properties can be functionalized with target-specific bacterial components, facilitating increased intracellular AMP uptake, such as ligand-functionalized nanoparticles that bind bacterial lectins or porins for direct internalization [17], thereby overcoming some of the intrinsic resistance caused by the Gram-negative outer membrane [15]. Nanoparticles, such as metallic nanoparticles, can disrupt bacterial membranes either independently or synergistically, along with AMPs thereby amplifying microbial damage such as silver nanoparticles, in combination with AMPs, can destabilize the outer bacterial membrane more effectively [15, 17].

Incorporation of AMPs within nanoparticles may be able to bypass antibiotic efflux pumps by preventing recognition and efflux, thereby favouring direct intracellular delivery [21]. Certain nanocarriers can encapsulate efflux pump inhibitors along with AMPs, ensuring higher intracellular AMP retention time within the microorganisms [26]. Co-encapsulation of antimicrobial adjuvants within lipid-based nanosystems (chitosan nanoparticles, nanostructured lipid carriers (NLCs)) prevented premature drug inactivation, fusogenic properties facilitated targeted delivery with enhanced biodistribution, and pharmacokinetic profiles [66]. Distinctive characteristics (including advantages and disadvantages) of different nanocarriers, which can be used as alternative novel drug delivery approaches, are summarized in the table (Table 2).

**Table 2:** Universal characteristics, advantages, and disadvantages of different nano-carrier systems

Nanocarrier	Key features	Advantages	Disadvantages
*Organic Nanocarriers*			
Polymeric material nanocarriers [67–70]	• Possess biodegradable polymers • AMPs can be dispersed within the matrix or functionalized at the nanocarrier’s surface	• Slow and sustained AMP release • Higher loading capacity • Selected and targeted delivery via ligand functionalization	• Complex manufacturing procedures and scale-up challenges • Potential cytotoxicity from polymer-degraded products
Lipid nanocarriers [67–69]	• Amphiphilic phospholipid bilayer vesicles having an aqueous core • Encapsulates hydrophilic and hydrophobic AMPs	• Slow and sustained AMP release with membrane-fusion–mediated delivery • Reduce cytotoxicity • Biocompatible • Surface modification (PEGylation)	• Low circulation half-lives • Instability and rapid clearance by the RES • Low circulation half-lives • Low drug loading capacity • Leakage of encapsulated drugs • Immunogenicity
Peptide-Dendrimer Hybrids [67–69]	• Branched, monodisperse dendritic scaffolds conjugated with multiple AMP branches	• Protease-resistant • Low haemolytic activity • High efficacy	• Immunogenicity • Complex manufacturing procedures and scale-up challenges
Cyclodextrin nanocarriers [67–69]	• Macrocyclic oligosaccharides with hydrophobic cavities form inclusion complexes for AMPs	• Enhance solubility and stability • Protect from proteolysis • Improved bioavailability • Biofilm penetration	• Limited loading capacity • Premature payload leakage • Variable biocompatibility
Nucleic acid nanosystems [67–69]	• Programmable DNA, RNA structures, aptamers, origami, and tetrahedral frameworks with AMP conjugation	• High specificity via aptamer targeting • Stimuli-responsive release • Precise structural control • Biocompatibility	• Undergo nuclease degradation • Immunogenicity • Complex design and synthesis
Self-assembled AMPs [67–69]	• AMPs are engineered to form nanostructures via non-covalent interactions (e.g., micelles, hydrogels)	• No exogenous carrier is needed • Stimuli-responsive disassembly • Highly biocompatible • Intrinsic antimicrobial function	• Low reproducibility • Unclear pharmacokinetics • Environmental sensitivity (pH, ionic strength
*Inorganic Nanocarriers*			
Metallic nanoparticles [70–73]	• Gold (Au), silver (Ag), or zinc oxide nanoparticles can be functionalized with AMPs • Exhibit intrinsic bactericidal activity • Rigid inorganic frameworks, either with pore sizes or layered structures	• Synergistic bactericidal effects due to ROS generation and membrane disruption • Multimodal uses such as imaging and therapy • High AMP loading capacity and stability • Stimulus-triggered response	• Potential cytotoxicity • Tendency to aggregate within the biological medium • Metal toxicity • Unstable within physiological conditions • Poorly biodegradable • Challenging clearance pathways • Complex synthesis

### Organic nanocarriers

Organic nanocarriers typically range from 1–1000 nm in size, can be formed from synthetic or natural organic compounds such as lipids, proteins, and polymers, are biodegradable, biocompatible, and able to incorporate different therapeutic agents [32]. Organic material nanocarriers incorporating AMPs can be divided into two broader categories: those with encapsulated AMPs and those with conjugated AMPs [33]. The former entraps AMPs within a carrier matrix, while the latter involves AMPs in the structure of organic materials [33]. Polymers, lipids, and nucleic acids are commonly used materials [33]. Notably, the organic nanocarriers can modulate the release profiles of AMPs, facilitate controlled and sustained drug delivery, and enhance antimicrobial efficacy while minimizing potential cytotoxic adverse events [32, 33]. Organic material nanocarriers that incorporate AMPs can be classified into various subtypes:

### Polymeric material nanocarriers

Polymeric material nanocarriers have provided novel routes for delivering AMPs against Gram-negative bacteria [74]. Polymeric nanoparticles are commonly composed of biodegradable and biocompatible polymers such as poly(ethylene glycol) (PEG), poly(lactic acid) (PLA), and poly(lactic-co-glycolic acid) (PLGA)-based copolymers that encapsulate AMPs for controlled and sustained release, prolonging the half-lives and maintaining therapeutic concentrations for extended periods, thereby reducing dosing frequency and systemic side effects [75–77].

The inherent versatility of polymers facilitates precise tailoring of particle size, surface charge, and functionalization with selected and targeted ligands, as well as favouring enhanced accumulation at bacterial infection sites and improving penetration through the outer membranes of Gram-negative pathogens [32, 33]. Moreover, they permit the incorporation of stimuli-responsive elements, such as pH or temperature-sensitive linkers, which can trigger AMP release in response to the acidic microenvironment at the infection’s site, thereby enhancing their site-specific release and therapeutic efficacy [75, 78].

PLGA nanoparticles loaded with AMPs have sustained release and enhanced activity against *K. pneumoniae* and *E. coli*, whereas chitosan-based nanoparticles can improve AMP penetration within the biofilms formed by *P. aeruginosa* [75, 76, 79]. Polymeric micelles and nanogels encapsulating AMPs can self-assemble into different nanostructures, enhancing membrane-disruptive properties, thereby increasing bacterial uptake, and potentiating their bactericidal effects [32, 33].

Functional modifications, such as conjugation with specific antibodies, zwitterionic or PEGylated surface engineering, stimuli-responsive linkers, quorum-sensing inhibitors, and pathogen-selective targeting (i.e., LPS-binding motifs, mannose ligands, aptamers) could improve selectivity towards Gram-negative bacteria, reducing off-target toxicity and overcoming multidrug resistance trends [33]. Polymeric nanocarriers, such as PEGylated chitosan and block copolymers, have facilitated sustained release with targeted biodistribution of AMPs, showing enhanced tissue retention in *P. aeruginosa* infections [19]. Polymeric AMP nanocarriers have also demonstrated improved stability within a protease-rich microenvironment [19].

The encapsulation efficiency of AMPs remains a key limitation, as a significant amount of peptide can be lost during solvent-based nanoprecipitation due to solvent exposure and phase partitioning, resulting in lower loading efficiency [80, 81]. To address these concerns, multi-framework approaches such as quality-by-design, supported experimental design and optimization, and continuous-flow microfluidic methods can help to give reproducible nanoparticle assemblies with better encapsulation [80, 81]. Moreover, mechanistic modelling and analytical processing technologies can facilitate regulatory-aligned and predictable translation of nano-peptide therapeutics [80, 81].

### Lipid nanoparticles

Lipid nanocarriers, such as liposomes, are spherical vesicles with a phospholipid bilayer that can incorporate both hydrophilic and hydrophobic compounds, improving AMP stability by protecting against enzymatic degradation and facilitating selective and targeted delivery to bacterial cells by enhancing fusion with bacterial membranes, thereby reducing host toxicity by minimizing non-specific interactions with circulating proteins and immune cells [82]. Lipid nanocarriers can be engineered with cationic lipids to enhance electrostatic interactions with the anionic outer membrane of Gram-negative bacteria, improving bacterial uptake and antimicrobial efficacy [82]. These nanocarriers may fuse with bacterial membranes, facilitating AMP delivery directly into cells [82].

Functionalization with targeting ligands or PEGylation can optimize liposomal formulations, reduce premature systemic clearance, and increase specificity for bacterial infections [82]. These physicochemical properties make lipid-based AMP delivery a promising alternative to conventional antibiotics, especially against multidrug-resistant (MDR) Gram-negative infections [82]. LL-37-loaded lipid nanoparticles have improved AMP stability and reduced inflammation in lung infections, and colistin-loaded liposomes have demonstrated increased antibacterial activity against *P. aeruginosa* [83].

In in-vitro studies, bio-inspired liposomes-conjugating peptides demonstrated enhanced antibacterial activity compared to free peptides [84]. Tuning liposomal charge and peptide density improved targeted binding to microbial membranes and reduced MICs against *P. aeruginosa* and *E. coli* [84]. Furthermore, liposomal hydrogel dressings encapsulating novel peptides (i.e., BrSPR-20-P1) improved drug release kinetics, with lower cytotoxicity, and stronger inhibition of bacteria within wound infections, demonstrating that liposomes can be used to bridge the clinical transition of topical Nano-AMPs [84].

Despite their therapeutic potential, lipid-based Nano-AMP delivery systems face various critical challenges. One major limitation is the instability of lipid nanoparticles within physiological conditions, where interactions with serum proteins, immune cells, and enzymatic degradation lead to premature leakage of encapsulated AMPs [83]. Furthermore, the potential toxicity of cationic lipid nanocarriers, while improving bacterial targeting, may disrupt mammalian cell membranes and induce host cytotoxic effects [83]. By addressing these concerns, lipid nanocarriers could emerge as an alternative innovative strategy for combating Gram-negative bacterial infections.

### Niosomes

Niosomes are self-assembled vesicular systems composed of cholesterol and non-ionic surfactants, providing biocompatible and stable platforms for delivering AMPs against Gram-negative bacteria [70, 85]. Their amphiphilic architecture favours the dual encapsulation of hydrophobic adjuvants and hydrophilic peptides [70, 85]. In contrast, the surfactants, such as those with varying alkyl-chain lengths and hydrophilic-lipophilic balances, can influence their physicochemical properties, thereby supporting membrane stability and release kinetics [70]. Surface modification strategies, such as PEGylation and charge-modifying additives, further prolong circulation half-life by limiting opsonization and uptake by RES [70].

Monolaurin-based niosomes, co-encapsulating polymyxin B, showed profound microbial membrane disruption and up to 9-log reductions in *A. baumannii*, *K. pneumoniae*, and *P. aeruginosa,* resulting in better bacterial clearance, because of lipid–peptide synergistic interactions and improved in-vivo biodistribution [86]. Niosomes functionalized with alginate and gelatin co-loaded with α-pinene and β-sitosterol demonstrated better efficiency and bactericidal activity against *carbapenem-resistant K. pneumoniae* [87]. Nano-encapsulation reduced cytotoxicity with significantly lower MICs at sub-therapeutic concentrations due to synergistic disruption of Gram-negative membranes [87].

Niosomes co-encapsulating LL-37 and lysostaphin yielded good physicochemical stability at 4 °C, preserving drug integrity without chemical alteration [88]. Higher entrapment efficiency favoured sustained antimicrobial action over 72 h against *A. baumannii* and *E. coli*, improving local drug bioavailability, showing dual drug niosomal systems can be used to deliver AMPs for managing chronic and life-threatening Gram-negative infections [88].

Niosomes can be relatively more stable (physical and chemical), have lower cytotoxicity, and better biocompatibility and biodegradability compared to liposomes [89, 90]. However, niosomes face certain challenges, such as scalability and cost-effectiveness, which rely on preparatory methods and surfactant-dependent features that may hamper vesicle fusion and storage, requiring careful optimization for clinical translation [89, 90].

### Solid lipid nanoparticles

Solid lipid nanoparticles (SLNs) (that remain in solid states either at room and body temperatures) composed of solid lipids (fatty acids, steroids, and TAGs), stabilized by surfactants and emulsifiers, can shield AMPs from enzymatic degradation [91–93]. Certain lipid matrices can undergo structural rearrangements to release the encapsulated drugs, can be controlled to achieve stable β transitioning forms, and this can directly influence peptide leakage and encapsulation efficiency. This highlights that lipid selection and cooling rates are critical parameters to modulate their physiochemical features [91–93].

Stearic-acid SLNs encased LL-37, achieved > 70% encapsulation efficacy, favouring sustained peptide release, and demonstrated potent activity against *E. coli* and *K. pneumoniae,* with reduced cytotoxicity, due to improved surface charge interactions and better penetration within the extracellular matrix of Gram-negative bacteria outer membranes [94, 95]. SLNs encapsulated polymyxin B achieved > 90% loading efficiency, because crystalline lipid matrix modulated drug release and maintained potency against resistant *P. aeruginosa*, improving bioavailability and potentially limiting systemic adverse events without affecting antimicrobial efficacy [96]. Limitations include scale-up challenges due to polymorphic transitions and homogenization, premature nanocarrier bursting during lipid crystallization, and drug expulsion during storage [95].

### Nanostructured lipid carriers

Nanostructure lipid carriers (NLCs) are advanced generations of lipid nanocarriers, incorporating both liquid and solid lipids to form a matrix with improved drug-loading capacity and reduced crystallinity over SLNs. Their partially disordered matrices prevent premature drug expulsion, favouring high drug entrapment efficiency, long-term stability, and sustained release [97, 98]. Surface modifications, i.e., incorporating cationic surfactants and targeting ligands, can be incorporated to enhance NLCs' interactions with pathogens, facilitating internalization within the target cells, thereby enhancing the intracellular delivery of therapeutic AMPs [97, 98].

NLCs co-loaded with cationic peptides and vancomycin demonstrated an eightfold MIC reduction against *E. coli*, triggered microenvironments-stimulant release, alongside significantly reduced inflammatory cytokines and in-vivo bacterial clearance, and reduced systemic toxicity [99, 100]. NLCs' surface can be engineered with alkaline phosphatase–responsive charge reversal, for dynamic drug control [100].

Anionic NLCs cores were sequentially coated with phosphate and protamine-based layers underwent enzyme-responsive charge inversion upon ALP and phosphate exposure, enhancing cellular internalization while mitigating cytotoxicity, highlighting that enzyme-triggered NLCs can be used as one of the rational strategies to improve biocompatibility and intracellular delivery of colloidal drug carriers [100–102]. However, NLCs face scale-up issues, nanocarrier aggregation under physiological ionic conditions and lipid oxidation variability over time [101, 102].

### Peptide-dendrimer hybrids

Peptide-dendrimer hybrids (PDHs) have emerged as novel nanocarrier routes for AMP delivery, offering innovative approaches for combating antimicrobial resistance (AMR) in Gram-negative bacteria [103]. These hybrid structures comprise of high-density branched architecture of dendrimers conjugated with bioactive AMPs or other targeting moieties, thereby enhancing their stability, local concentration at the microbial surfaces, and antimicrobial efficacy [103]. The dendrimeric scaffold provides a protective environment, preventing proteolytic degradation and reducing off-target cytotoxic events [103].

PDHs demonstrate synergistic antibacterial effects, disrupting bacterial membranes, thereby facilitating intracellular AMP penetration, the key factor for resistance against Gram-negative pathogens [103]. Moreover, the functionalization of PDHs either with hydrophobic moieties or cationic molecules can enhance their interaction with anionic bacterial outer membranes, favouring membrane destabilization and bacterial cell death [103]. AMP-dendrimer hybrids have shown higher antimicrobial activity compared to free AMPs against *P. aeruginosa*, while dendrimer-based Nano-AMPs have demonstrated an improved therapeutic index with reduced host cytotoxicity [103].

Multivalent peptide dendrimers such as G3KL/T, which feature protease-resistant scaffolds, enhance AMP membrane disruption by improving serum stability and biofilm penetration. These properties are particularly effective against *A. baumannii* and *P. aeruginosa*, enabling rapid bacterial killing with minimal resistance development and demonstrating strong potential against chronic Gram-negative infections [104, 105]. Mechanistically, increased cationic charge and molecular branching enhance binding affinity to LPS-rich outer membranes, expanding the design space while maintaining dendrimer suitability for Nano-AMP formulations [104, 105].

Despite their promise, several key challenges must be addressed to translate the clinical potential of peptide dendrimer hybrids (PDHs). The complexity of peptide-dendrimer synthesis and optimization presents significant formulation hurdles [103, 106]. Moreover, their cationicity can trigger complement activation and increase the risk of hemolysis [103, 106]. To mitigate immunogenicity and enhance safety, strategies such as charge tuning, zwitterionic surface coatings, and PEGylation should be explored [103, 106]. Preclinical studies have primarily focused on Gram-negative pathogens, and the modular chemistry of dendrimers makes them well-suited for pairing with lipid or polymeric nanocarriers to reduce cytotoxicity [103, 106]. Therefore, careful consideration must be given to refining PDH designs via structural modifications that can optimize therapeutic efficacy and safety [103, 106].

### Cyclodextrin nanocarriers

Cyclodextrin (CD) nanocarriers can enhance the specificity and efficacy of AMP delivery, offering improved solubility, stability, and targeted release [107–109]. Cyclodextrins are cyclic oligosaccharides having a hydrophobic inner cavity with an outer hydrophilic surface, allowing them to encapsulate AMPs, protecting them from enzymatic degradation, enhancing their solubility in physiological environments, and facilitating controlled peptide release at the infection site [107–109]. Moreover, cyclodextrin nanocarriers can be engineered to respond to specific environmental triggers, such as pH, redox gradients, or enzyme-degradable linkers, ensuring AMP release preferentially within the infected tissues [107–109].

Cyclodextrin-based nanocarriers can enhance the antimicrobial activity of AMPs by improving their interaction with microbial membranes [107, 109]. Certain modified cyclodextrins, such as amphiphilic cyclodextrins, can integrate within the bacterial lipid bilayers, thereby facilitating AMP insertion and amplifying the membrane disruption [107–109]. Moreover, they have demonstrated synergistic effects when co-delivered with conventional antibiotics or metal nanoparticles by increasing peptide retention time at infection sites to combat chronic infections caused by multidrug-resistant (MDR) Gram-negative bacteria [107–109].

β-Cyclodextrin (β-CD), a cyclodextrin derivative, forms inclusion complexes that can be cross-linked into nanoparticles, enhancing solubility, protecting encapsulated cargo, and enabling sustained release of AMPs, and so addressing their typically short half-life [110]. Cyclodextrin-based nanogels and microgels can have improved antibacterial activity, with emerging research on cross-linked and PEG-stabilized β-CD nanoparticles highlighting their biodegradable and biocompatible properties [110]. Furthermore, cyclodextrin nanocarriers can co-deliver peptide motifs that potentiate quinolone activity against *E. coli* and *P. aeruginosa*, suggesting that hybrid nanocarrier strategies may reduce required antibiotic dosages by enhancing drug sensitivity [111].

Despite their promising advantages, optimization of cyclodextrin-based nanocarriers and potential immunogenicity still need to be addressed for clinical translation [110]. At present, they are less standardised for parenteral use, and Gram-negative targeting might require ligand functionalization, such as using LPS binders, to improve therapeutic outcomes [110].

### Nucleic acid nanosystems

Nucleic acid nanosystems are a promising, innovative approach for enhancing the specificity and efficacy of AMP delivery by conjugating them with nucleic acids such as RNA, DNA, or aptamers, improving stability and pharmacokinetics, thereby overcoming key limitations associated with conventional therapies [112–115].

Conjugation of AMPs with nucleic acids can be achieved through electrostatic interactions, covalent bonding, or nanocarrier encapsulation, enabling precise targeting of bacterial pathogens [113–115]. Aptamers are short single-stranded RNA or DNA molecules having high binding affinity for microbial surface markers, and can selectively bind AMPs to resistant Gram-negative bacteria, minimizing off-target effects and reducing host cytotoxicity [112–115]. Apart from providing structural support for AMP conjunction, nucleic acid-based nanocarriers also facilitate stimuli-responsive release mechanisms, triggering peptide activity in response to specific microbial environments, such as acidic pH, redox gradients, or enzymatic degradation [112–115].

Beyond enhancing selective and targeted binding, nucleic acid nanosystems can improve bioavailability and stability by protecting them from enzymatic degradation and premature systemic clearance [113–116]. Incorporation of nucleic acids within the nanoscale delivery systems has multifunctional therapeutic potentials, such as co-delivery of AMPs along with gene-silencing molecules, small interfering RNA (siRNA), or antisense oligonucleotides can amplify antimicrobial activity by suppressing bacterial resistance genes [113–116]. Moreover, CRISPR-based nucleic acid nanosystems have emerged as an innovative approach for selectively eradicating resistant bacteria by combining CRISPR-Cas gene-editing tools with AMPs to disrupt key essential bacterial genes [114–116]. Recent preclinical studies, such as NANs delivering LL-37 analogues to infections, underscore the therapeutic potential to redefine precision peptide therapeutics in antimicrobial contexts [114, 115].

Nano-carriers incorporating DNA and RNA (and hybrids) have demonstrated improved stability and effective cellular interactions, making them promising platforms for AMP encapsulation, particularly in treating Gram-negative infections [117]. However, several barriers remain, including high manufacturing costs, limited serum stability, challenges with endosomal escape, and potential immunostimulation. Recent analyses have mapped these bottlenecks, suggesting that design automation and chemical stabilization could enhance clinical outcomes [117]. For targeting Gram-negative pathogens, nucleic acid nanocarriers can offer precision by incorporating aptamers and anti-LPS ligands, which improve binding specificity and allow for lower therapeutic doses while effectively managing infections [118].

### Self-assembled AMPs

Self-assembled AMPs are usually formed through various interactions such as intermolecular hydrogen bonding, hydrophobic interactions, ionic-complementary forces, aromatic stacking, and electrostatic interactions, enabling AMPs to spontaneously assemble into nanostructures such as vesicles, micelles, nanotubes, or nanofibers in aqueous environments [119–124].

Different self-assembly approaches allow the formation of hierarchical structures that achieve a minimum energy state, leading to enhanced stability and functionality of the peptides [119–124]. Moreover, the self-assembly process can be optimized by modifying AMP structural sequences or incorporating co-assembling molecules such as lipids or polymers, thereby enhancing their physicochemical properties and bactericidal activity [119]. The self-assembly of AMPs results in ordered nanostructures capable of effectively interacting with bacterial membranes, thereby improving their antimicrobial activity [119].

Certain AMP-based nanostructures demonstrate enhanced membrane disruption capabilities because of their ability to form pore-like structures upon interaction with bacterial lipid bilayers [119]. Co-assembly with biocompatible polymers, such as chitosan or polyethylene glycol (PEG), can enhance peptide solubility, improving therapeutic efficacy while reducing potential host cytotoxicity [119]. Future research should focus on optimizing AMP self-assembly mechanisms and conducting preclinical and clinical studies to evaluate their efficacy and safety to combat antimicrobial resistance.

Self-assembled Nano-AMPs are designed to form different nanostructures, such as micelles, nanofibers, and nanotubes, respectively, enhancing stability, protecting against proteolysis, and enabling stimuli-responsive release under infection micro-environments [125, 126]. N6-based constructs can be transformed from nanoparticles to nanofibers in the presence of microorganisms, which improves in-vivo stability and efficacy against *E. coli* and *P. aeruginosa* [125, 126].

However, self-assembled nano delivery approaches bring several challenges, such as aggregation that can alter pharmacokinetic parameters, i.e., absorption, distribution, clearance, alongside off-target interactions, which can increase cytotoxicity [127]. AI-driven sequence optimization enables precise tuning, amphipathic balance, and cytotoxicity, which may accelerate the development of self-assembling AMPs with improved biocompatibility and safety [128, 129].

### Inorganic nanocarriers

Inorganic nanocarriers demonstrate distinctive features, making them exceptionally well-suited for diagnostic and therapeutic purposes [73]. Their surface properties can be engineered via shell growth and ligand exchange, such as targeting ligands, PEG coatings, and incorporation of responsive moieties, thereby favouring better biodistribution, circulation half-lives, and higher cellular uptakes [73].

Inorganic material nanocarriers incorporating AMPs can be divided into either metallic nanoparticles or nanotubes [73]. Nano-drug delivery systems utilize non-covalent interactions, such as hydrophobic interactions and ionic-complementary forces, to stabilize AMPs, improving their pharmacokinetic profiles and prolonging in-vivo activity [73]. Nanocarriers, such as metallic nanoparticles (gold, silver, zinc oxide), mesoporous silica nanoparticles (MSNs), and layered double hydroxides (LDHs), can provide structural support and protect AMPs from enzymatic degradation, prolonging their bioactivity within the physiological conditions [73].

## Metallic nanoparticles

Metallic nanoparticles have gained significant attention in recent years as an alternative route for delivering AMPs, particularly against Gram-negative bacterial infections, where conventional antibiotics often falter [130–132]. Moreover, their unique physicochemical properties, such as ease of functionalization with modified surface properties, high surface area-to-volume ratios, inherent bactericidal effects, enhanced bioavailability, biocompatibility, and targeted delivery of AMPs [130–132]. Encapsulation within metallic nanoparticles, such as gold, silver, or zinc oxide, protects AMPs from rapid enzymatic degradation and off-target effects and facilitates sustained and controlled release at the infection site [130–132].

Metal nanoparticles exhibit intrinsic antibacterial effects while providing a stable delivery platform, thereby enhancing the overall therapeutic effect [130, 131]. Functionalization of inorganic nanocarriers with surface modifications or targeting ligands allows for controlled AMP release, reducing off-target effects with minimal host cytotoxic events [130, 131]. Incorporation of AMPs within the layered double hydroxides (LDHs) can offer sustained drug release kinetics, as LDH structures degrade within the bacterial microenvironments [131, 132]. These drug-delivery approaches are developed to address the limitations of free AMPs, such as poor pharmacokinetics and high cytotoxicity [131, 132]. Therefore, conjugating AMPs within the inorganic nanoparticles can improve their overall therapeutic potential against resistant pathogens by enhancing their stability, thereby reducing toxicity [130–132].

Synergistic antimicrobial effects between metallic nanoparticles and AMPs have demonstrated enhanced membrane disruption due to metallic nanoparticle interaction with bacterial surfaces, overcoming bacterial resistance by targeting multiple biochemical pathways [133–135]. Silver nanoparticles combined with polymyxin B have demonstrated enhanced killing of Gram-negative pathogens (such as *A. baumannii* and *P. aeruginosa*), and gold nanoparticles loaded with AMPs have improved stability and biofilm penetration [133–135]. Zinc oxide and titanium dioxide nanoparticles generate reactive oxygen species (ROS) upon interaction with microbial cells, further potentiating AMP antimicrobial activity [130–132].

Despite these promising advantages, the major concerns with metallic nanoparticles are their potential intrinsic cytotoxicity, induction of oxidative stress, uncontrolled ion release, and protein corona formation, which can alter cellular uptake, off-target interactions with opsonin proteins, and immunogenicity [133–135]. Variability in nanoparticle synthesis can also lead to inconsistency in particle sizes, surface charges, and nanoparticle aggregation, leading to inconsistent release kinetics [136].

Long-term tissue accumulations, especially liver, kidneys, and spleen, highlight the potential for acute clearance and chronic toxicity [136]. Due to these regulatory and safety concerns, the clinical transition to the systemic use of metallic nanoparticles is closely scrutinised. Therefore, controlled passivation approaches, such as surface coatings and local delivery, e.g. inhalation, implants, and wound dressings, stand out as pragmatic routes toward safer topical antimicrobial clinical translation [136].

### Mesoporous silica nanoparticles

Mesoporous silica nanoparticles (MSNs) are being used as next-generation nano-AMPs due to their larger surface area and porous structure, which offer higher loading capacity while preserving structural integrity, enabling the encapsulation of AMPs with precise drug release kinetics in response to microenvironment stimuli [130, 131]. Generally, silica is regarded as safer; however, biocompatibility is dependent on size, surface chemistry, and doses, emphasizing the importance of surface functionalization and rational designs for therapeutic uses [137, 138]. Moreover, MSNs can be chemically modified to reduce macrophage activation and to achieve sustained AMP release, essential for managing infections caused by Gram-negative pathogens [139, 140].

RGD-functionalized MSNs encased LL-37, preventing implant-related infections by Gram-negative bacteria, particularly *E. coli* [139]. MSNs encapsulated LL-37 demonstrated better antimicrobial potency, while RGD functionalization modulated macrophage pyroptosis to maintain immune competence and reduce inflammation [139]. In a murine implant model, MSNs loaded with LL-37 improved wound healing and infection clearance, demonstrating better biocompatibility and efficacy compared to unmodified MSNs [139].

MSNs loaded with HHC36, engineered to bridge with diselenide and grafted onto titanium implants for orthopaedic applications prone to *E. coli* and *P. aeruginosa* infections [140]. Linking with diselenide, it provided oxidation-dependent AMP release within the inflammatory microenvironment, aligning with antimicrobial actions [140]. in-vitro studies showed > 95% bacterial killing, while in-vivo osteomyelitis model showed minimal inflammatory infiltrations and promoted improved osseointegration by eradicating bacterial loads, demonstrating MSN nano-coatings can serve as dual antimicrobial and immunomodulatory properties to manage the infection dynamics [140].

## Therapeutic applications of Nano-AMPs

### in-vitro trials

In several pre-clinical studies, Nano-AMPs (i.e., organic and inorganic nanocarriers) have emerged as promising alternative therapeutic strategies, having effective efficacy and safety profiles in comparison to conventional antimicrobials (Tables 3 and 4) [46, 75, 76, 83, 137, 141–145, 147–153, 161, 162, 164–169, 171, 172, 175–181]. in-vitro studies have demonstrated that Nano-AMPs employing different nanocarrier approaches, such as liposomes, polymeric nanoparticles, and inorganic nanostructures, have significantly improved peptide stability, bioavailability, and biocompatibility [137, 147, 175–177].

**Table 3:** Characteristics and key findings of different in-vitro trials using organic nanocarrier systems

AMPs *n* = 41	Nanocarriers	Target Pathogens	Key Findings
KYE28	Self-assembly	*E.C*	Increasing antimicrobial stability [120]
Thermo-responsive chitosan (TCTS)	Self-assembly	*A. B*	Enhance antimicrobial and anti-inflammatory, favour wound healing and neovascularization [121]
LL-37	Self-assembly	*E.C*	Enhance antimicrobial and anti-inflammatory, favour chronic wound healing and angiogenesis [122]
PA-4 and PA-7	Self-assembly	*K.P*	Micelle-forming PAs had an excellent antimicrobial activity with increased cell membrane permeability and disruption of the pathogen’s membrane, leading to cell lysis and death [141]
D-W362	Self-assembly	*E.C*	SAANs, supramolecular assemblies of AMPs, underwent programmed self-assembly into nanostructured fibres to “punch holes” in the bacterial membrane, thus killing them [142]
Bacitracin and gramicidin	Self-assembly	*E.C* *P. A*	Broad-spectrum antibacterial activity with less toxicity after self-assembly into nanofiber structures [143]
Polyoxometalate (L1)	Self-assembly	*E.C*	Multivalent peptide nanofibers underwent self-assembly with concentrated positive charges and were excellent multivalent ligands for binding with bacterial cells [144]
ASCP1 and ASCP2	Self-assembly	*E.C*	When exposed to external stimuli, these underwent an abrupt structural transition from a random coil to a stable unimolecular β-hairpin conformation, forming an elastic hydrogel [145]
CT9W_1000_	Self-assemble micelles	*P. A*	Higher stability under salt serum, ions, and acid–base environments, and is highly resistant to trypsin degradation [123]
MH5C coupled to polymers (PEG)	Polymers peptide	*E.C* *P. A*	Inhibited bacterial growth [146]
Nisin-GE	Lipid nanocarriers	*E.C*	Differences of 3–4 log CFU/ml in viable counts, synergistic effects to overcome stability issues [147]
WLBU2	Lipid nanocarriers	*P. A*	Enhanced antimicrobial activity and effective PACT efficiency against Gram-negative pathogens [148]
ParELC3	Lipid nanocarriers	*E.C*	Enhanced antimicrobial activity and bioactivity with no cytotoxic effects [149]
Polymyxin B	Lipid nanocarriers	*E.C* *P. A*	Killing kinetics revealed total cell death at 12 and 24 h for *P. aeruginosa* & *E. coli* [150]
Colistin	Lipid nanocarriers	*P. A*	Increased antimicrobial activity, drug release kinetics, and no cytotoxic events [151]
Colistin	Lipid nanocarriers	*P. A*	Theragnostic, colistin-encapsulated liposomes were effective for imaging and treating infections [83]
Colistin	Lipid nanocarriers	*P. A*	Superior antibacterial activity against clinical isolates [152]
Polymyxin B	Lipid nanocarriers	Gram-negative pathogens	Enhanced antimicrobial & penetration activity [153]
Polymyxin B	Lipid nanocarriers	*P. A*	Effective bactericidal activity against resistant strains [96]
LL-37 loaded with lysostaphin	Niosomes	*A.B,* *E.C*	Stable vesicles with prolonged antibacterial activity and good storage stability, i.e., 4 °C, for 2 months, supporting sustained local exposure with lower peaks & longer coverage [88]
Nisin & EDTA (LPS-permeabilizer)	Niosomes	*E.C*	Nisin retained antibacterial activity when combined with EDTA, highlighting niosomes as membrane-sensitization-assisted AMP delivery [154]
LL-37	SLNs	*P. A*	Preserved epithelial barrier functions, with better activity at reduced doses [155]
Polymyxin B	SLNs	*P. A*	Polymyxin B retained antibacterial efficacy with improved formulation stability [96]
Polymyxin B	SLNs crosslinked with alginate	*P. A*	Cross-linked SLNs were associated with reduced cytotoxicity & better antimicrobial activity [156]
Colistin sulfate	SLNs	*P. A*	Improved pulmonary delivery & therapeutic index for CF-related infections [157]
Colistin sulfate	SLNs	*P. A*	Showed better bactericidal activity [158]
Colistin	SLNs	*P. A*	Showed better bactericidal activity [159]
LL-37 + Serpin A1	SLNs	*E.C*	Co-delivery enhanced antibacterial efficacy & promoted wound closure [160]
Polymyxin B	Conjugated erythrocyte lipid nanocarriers	*E.C* *K. P*	Enhanced antimicrobial activity, effective & targeted delivery [161]
Colistin	Chitosan-lipid nanocarriers	*P. A*	Enhanced antimicrobial effects (fourfold) against clinical & resistant isolates [162]
Polymyxin B	Chitosan-lipid nanocarriers	*A. B*	Significant antibacterial effects [163]
Colistin	PLGA nanoparticles	*P. A*	Efficient entrapment, prolonged release of AMPs, penetrated biofilms and extended in-vitro anti-biofilm activity of colistin [164]
Nisin	Poly-(γ-PGA) & chitosan nanoparticle	*E.C*	Increased antimicrobial activity and stability [165]
e-Polylysine	Polymer-based nanofibers	*E.C*	Lower bacterial colonization with no cytotoxicity to human corneal epithelial cells [166]
Melittin	PEG nanocarriers	*E.C*	Antimicrobial effects, with extended drug release [167]
HHC10	PLGA-nanoparticles	*E.C*	In-vitro inhibition of bacterial growth, nontoxic to macrophage cells in vitro after encapsulation. Up to 91% cellular internalization within 24 h [168]
K4	PLGA-nanoparticles	*P. A*	Enhanced antimicrobial activity with improved wound healing and angiogenesis [169]
GIBIM-P5S9K	PLGA-nanoparticles	*E.C* *P. A*	Enhanced bactericidal activity [170]
MSI-78	PLGA-PEG-nanoparticles	*P. A*	Increased antimicrobial activity, i.e., MIC 8–16 μg mL − 1 and safety [76]
SAAP-148	Poly(lactic-co-glycolic) acid nanoparticles	*A. B*	Increased antibacterial activities (10–20 fold) [171]
PA-13	Chitosan Dextran sulfate	*P. A*	Improved stability & antibacterial activity within tyrosine-challenged conditions [172]

*A. B* Acinetobacter baumannii, *E.C* Escherichia coli, *K. P* Klebsiella pneumoniae, *P. A* Pseudomonas aeruginosa, *V.C.* Vibrio cholerae

**Table 4:** Characteristics and key findings of different in-vitro trials using inorganic nanocarrier systems

AMPs *n* = 14	Nanocarriers	Target Pathogens	Key Findings
Daptomycin	Mesoporous titania	*P. A*	Enhancing the loading capacity, antimicrobial activity, stability, and selectivity [173]
Polymyxin B	Mesoporous silica nanoparticles	Gram-negative pathogens	Stronger synergy and higher antimicrobial effect, with no cytotoxic effects [137]
Esculentin-1a	Gold nanocarriers	*P. A*	Enhanced antipseudomonal activity up to 15-fold without being toxic to human cells, and increased the peptide’s re-epithelialization activity [174]
koreensis DC4 strain,	Gold-silver nanocarriers	*E.C*	Silver nanoparticles have enhanced antimicrobial activity, while gold nanoparticles show catalytic activity [175]
1018K6 peptide	Gold nanocarriers	*S. typhi*	Enhanced bacterial killing at sub-micromolar concentrations [176]
LL37	Gold nanoparticle	Gram-negative pathogens	Enhanced antibacterial effects with less cytotoxicity & better angiogenic activity [75]
Esculentin-1a, Esc	Gold nanoparticle	*P. A*	Covalent conjugation of Esc to soluble AuNPs via PEG linker increased antimicrobial activity by 15-fold & promoted wound healing on a keratinocyte monolayer [174]
Nisin	Silver nanocarriers	*E. faecalis*	Enhance antimicrobial and catalytic activity [177]
Andersonin-Y1, CAY1, AY1C	Silver nanocarriers	*A.B, E.C* *P. A, S. typhi*	Increased bactericidal activity. MD simulations demonstrated pore formation within membrane bilayers, mediated via a hydrophobic collapse mechanism [46]
Nisin	Silver nanoparticles	*E.C., K.P* *P. A, S. typhi*	AgNPs with nisin inhibited gram-negative bacteria. Therefore, a wound dressing with antimicrobial activity may be developed [178]
KYE21 & WWWKYE21	Titanium dioxide nanoparticle	*E.C*	Bacteria- and lipopolysaccharide-like membrane attachment using peptide-enhanced antibacterial effects with selective toxicity [179]
LL-37	Titanium dioxide nanoparticle	*E.C*	Higher membrane attachment ability to anionic membranes compared to mammalian [180]
AS-48	Biomimetic magnetic nanoparticles	*A.B, E.C* *P. A*	Enhanced growth inhibition effects [181]
Ib-M2	Iron oxide nanoparticle coated with chitosan	*E.C*	Enhanced growth inhibition effects [42]

*A. B* Acinetobacter baumannii, *E.C* Escherichia coli, *E. faecalis* Enterococcus faecalis, *K. P* Klebsiella pneumoniae, *P. A* Pseudomonas aeruginosa, *S. typhi* Salmonella enterica serovar Typhi

in-vitro model, Nano-AMPs can disrupt microbial membranes and exhibit potent antimicrobial activity against a spectrum of pathogens, including multidrug-resistant Gram-negative [11]. Recent in-vitro trials have highlighted the synergistic effects of Nano-AMPs along with traditional antibiotics, minimizing host cytotoxicity due to controlled drug release kinetics, thereby mitigating the likelihood of developing resistance, particularly against Gram-negative pathogens [46, 182–186].

Gold nanoparticles (AuNPs) functionalized with the AMP melittin with or without cysteine in the presence of non-physiological concentrations of proteolytic enzymes demonstrated higher antimicrobial activity, stability in serum and lower cytotoxicity than soluble AMPs, within in-vitro and in-vivo models (chronic wound infection) [187]. Silver nanoparticles (AgNPs) conjugated to cysteine, nisin or lysine exhibited synergistic effects against Gram-negative pathogens, penetrating AMPs within microbial cells, thereby facilitating lysis and bacterial cell death (99% reduction in viability) by disrupting microbial membrane integrity [46, 182–186]. Co-encapsulation of AMPs along with antibiotics such as nisin along with colistin, and P10 with ceftazidime within nanocarriers demonstrated synergistic antimicrobial effects against MDR *A. baumannii* and colistin-resistant *P. aeruginosa* [188].

Chitosan-based nanocarriers loaded with cationic AMPs (Indolicidin and LL-37) enhanced the AMPs' stability against enzymatic degradation, promoting rapid cellular uptake, and reducing cytotoxicity compared to free peptides or alternative formulations [79, 189, 190]. In a 3D epidermis model, liposomal LL-37 effectively inhibited infection at high concentrations with no cytotoxicity, potentially enhancing its therapeutic window [189]. Chitosan-based nanoparticles, biosynthesized using Olea europaea leaf extract, demonstrated dose-dependent antibiofilm activity against Gram-negative pathogens, indicating promising potential, particularly for combating multidrug-resistant pathogens in immunosuppressed patients [187].

Gold nanoparticle-encapsulated LL-37 demonstrated broad-spectrum antimicrobial activity in human serum, with reduced cytotoxicity at supra-therapeutic doses [75]. These nano-immobilized configurations improved host compatibility and restricted antimicrobial actions, thereby overcoming major translational barriers associated with peptides coated on medical devices, highlighting immobilization can be used to fine-tune AMPs bioactivity at the bio–nano interfaces, while preserving their therapeutic functions [75].

in-vitro model, ionic-liquid–modified mesoporous silica nanoparticle using INT assay showing dose-dependent inhibition of *E. coli,* demonstrating that surface chemistry modulation can facilitate silica-based AMP targeted binding [125]. Parallel to inorganic modalities, self-assembled Nano-AMPs are engineered to facilitate aggregation-mediated bacterial entrapment, thereby enhancing in-vitro aggregation propensity, resulting in *E. coli* binding and killing, due to higher localized concentration and membrane disruption synergistic effects [126]. Therefore, nano-carrier characteristics and designs, such as charge, aggregation control, and pore gating, can hamper the in-vitro efficacy of nano-AMPs against Gram-negative pathogens [126].

Nanocarriers with enzyme-responsive controlled release properties, conjugated with target enzymes (proteases or phosphatases) or membrane-damaging peptide derivatives (temporin L), converted the inactive peptides into active forms having membrane-damaging properties, thereby facilitating controlled and sustained drug release within the stimulus-responsive microenvironment [78]. Liposomal formulations encapsulating polymyxin B (cyclic AMP analog) bypassed efflux-mediated resistance in *E. coli* by facilitating direct fusion with the bacterial membranes and showed a significant reduction in bacterial growth after treatment with AuNPs containing polymyxin B, up to a sixfold decrease in MICs when comparing colistin alone, thereby showing synergism between polymyxins and AuNPs against *E. coli* [191, 192].

## In-vivo trials

Several pre-clinical in-vivo trials have substantiated the therapeutic potentials of different organic and inorganic Nano-AMPs (Tables 5 and 6) [38, 39, 45, 48, 49, 51, 138, 146, 174, 179, 193–200, 202–211, 213–215]. Electrostatically encapsulated liposomal colistin demonstrated sustained drug release, better tissue retention, and enhanced bactericidal activity against *P. aeruginosa*, while limiting peak systemic concentrations, supporting targeted pulmonary drug delivery with lower cytotoxicity [199]. This nano-carrier delivery method has illustrated how intralipid nanoparticles' electrostatic stabilization can improve therapeutic index without hampering bactericidal activity [199].

**Table 5:** Characteristics and key findings of different in-vivo trials using organic nanocarrier systems

AMPs *n* = 28	Nanocarriers	in-vivo model	Target Pathogens	Key Findings
Polymyxin B	Lipid nanoparticles	Mouse model	*P. A*	Reduced pulmonary CFUs, with increased time retention of the antibiotics within the lung tissues [38]
Polymyxin B	Lipid nanoparticles	ND4 Swiss Webster mice	*P. A*	4.6–11.1-fold higher drug exposure in pulmonary tissues, reduced lung bacterial burden, with improved survival rates [39]
Polymyxin B	B-Polysaccharide-nanoparticles	Mice	*P. A*	Enhanced efficacy and safety [193]
Polymyxin B	Lipid nanoparticles	BALB/c mice	*A. B*	It targeted and disrupted bacterial cell membranes, generating increased ROS, & modulated wound healing by M1/M2 macrophage polarization and promoted angiogenesis, collagen regeneration and granulation tissue formation [194]
Polymyxin B co-loaded with monolaurin	Niosomes	Mice	*K.P*	Disrupted bacterial membranes by targeting LPS, enabling synergistic killing with polymyxin B, reported no cytotoxicity in-vitro & in-vivo [86]
FU002 derived from vancomycin	Lipid nanoparticles	Wistar rats	*E. faecium* & *faecalis*	Increased antimicrobial activity and bioavailability [195]
LL-37	Lipid nanoparticles	Rabbit	*E.C*	Promoted wound closure, reduced bacterial contamination, and enhanced anti-inflammatory activity with accelerated wound closure in fibroblast cells and keratinocytes [160]
S-thanatin (Ts)	Lipid nanoparticles	ICR mice	*K. P*	Strong synergistic effects restored the susceptibility against MDR pathogens, reduced the lethality rate of the septic shock, and resulted in rapid bacterial clearance in mouse models [196]
Colistin	Lipid nanoparticles	Mice	*E.C*	Reduced the bacterial burden by over 6-log reduction and alleviated inflammation [197]
Colistin	Lipid nanoparticles	C57BL/6 mice	*P. A*	Eliminated 99.99% of biofilm-embedded bacteria. Moreover, 99.7% reduction in bacterial colonization, and significantly mitigated inflammation and pulmonary fibrosis [198]
Colistin	Lipid nanoparticles	Kunming (KM) mice	*P. A*	Good entrapment efficacy, redistribution into bacterial cell membranes to exert bactericidal activity, and good retention time within tissues [199]
Colistin	Lipid nanoparticles	Sprague–Dawley rat	*E.C*	Increased entrapment efficiency with good retention times (fourfold increase in the plasma) [200]
RKKKRLLRKKC co-delivered with vancomycin	NLCs	Mice	*E.C*	Infection-enzyme–responsive destabilization, eightfold MIC reduction, & ninefold reduction in bacterial loads in-vivo [100]
Colistin sulfate	Chitosan-nanoliposomes	Mice	*E.C*	Improved localization and bioavailability within the thigh muscles of infected mice [201]
OH-CATH30	Carboxymethyl chitosan-nanoparticles	Kunming (KM) mice		Enhanced antimicrobial activity with better wound healing properties by increasing granulation tissue formation because of re-epithelialization and neovascularization [202]
Tet213	Polymers peptide	Sprague–Dawley rat	*E.C*	Favoured reepithelialisation, collagen deposition, and angiogenesis in infected wound animals [203]
Chitosan–mastoparan	Chitosan-based polymeric NPs	BALB/c mice	*A. B*	Significant reduction in bacterial CFUs, with good biocompatibility and cytocompatibility [204]
LL-37	PCL-based polymeric nanofibers	Rat	*P. A*	Antimicrobial wound dressing, with adequate cell infiltration, favours tissue regeneration [205]
SET-M33 synthetic peptide	Dextran-based single-chain polymer NP	BALB/c mice	*P. A*	Improved time-kill kinetics and lung residence time of the peptide [206]
APO	PVA-based polymeric nanofibers	Mice	*A. B*	APO monomer patches improved wound appearance, reduced wound size, and wound bacterial loaded accelerated wound healing [207]
Human cathelicidin peptide 17BIPHE2	PCL-based polymeric nanofibers	Mice	*A. B, K.P, P. A*	Strong antimicrobial activity. For the chronic wound model, 17BIPHE2-containing nanofiber dressings could eliminate the microbes [208]
Esculentin	PLGA nanoparticles	C57BL/6 J mice	*P. A*	Enhanced antimicrobial activity with 3-log reduction of pulmonary bacterial growth for up to 36 h [48]
LL-37	PLGA nanoparticles	Mice	*E.C*	Improved wound healing with better bactericidal activity [209]
Cathelicidin-BF	Nanocarriers (epigallocatechin-3-gallate & silk fibroin)	BALB/c mice	*E.C*	Increased antibacterial effects & LPS adsorption. in-vivo therapeutic effect against ulcerative colitis [179]
SET-M33	Poly(lactide-co-glycolide) conjugated with PEG	BALB/c mice	*P. A*	Enhanced penetration of artificial mucus and bacterial alginate by PEGylation. Sustained release and persistent antibacterial activity [210]
Poly (ethylene glycol) diacrylate/thiolated chitosan (TCS)	Self-assembly	Sprague–Dawley rat	Gram-negative pathogens	Enhanced antimicrobial and anti-inflammatory, favouring wound healing and neovascularization [121]
Trp-Arg-Trp-Arg-Trp-Tyr (WRWRWY)	Self-assembly	Mice	*E.C*	Enhanced antibacterial effects & boosted wound healing in mice skin [124]
T9W	Self-assembly	ICR mice	*P. A*	Enhanced antibacterial effects up to 1.5–4 times & reduced lung injury and pro-inflammatory cytokines [123]

*A. B* Acinetobacter baumannii, *E.C* Escherichia coli, *E. faecium & faecalis* Enterococcus faecium & faecalis, *K. P* Klebsiella pneumoniae, *P. A* Pseudomonas aeruginosa

**Table 6:** Characteristics and key findings of different in-vivo trials using inorganic nanocarrier systems

AMPs *n* = 9	Nanocarriers	in-vivo model	Target Pathogens	Key Findings
β-cyclodextrin	Mesoporous silica nanoparticles	Mouse	*P. A*	Increasing antimicrobial stability, preventing host tissue damage and inflammation [211]
T7E21R-HD5 with succinylated casein (SCN)	Mesoporous silica nanoparticles	Mice	*E.C*	Enhanced antimicrobial activity, non-cytotoxic, alleviated intestinal inflammation by suppressing the production of inflammatory factors TNF-α, IL-1β, and MMP-9 [138]
DNA aptamer	Gold nanocarriers	FvB mice	*S. typhi*	Increased host cell viability with 100% survival rates [212]
DNA aptamer	Gold nanocarriers	ICR mice	*A. B*	Marked reduction in bacterial colonization, with increased survival time and rate [45]
LL-7	Gold nanocarriers	Mice	Gram-negative pathogens	Enhanced bactericidal activity, promoted angiogenesis, and inhibited bacterial infection in diabetic wounds, accelerated wound closure rates, faster re-epithelization, improved granulation tissue formation, and high VEGF expression [51]
Cecropin-melittin	Gold nanocarriers	C57/BL6 mice	Gram-negative pathogens	Increased antimicrobial activity and stability [213]
C-At5	Gold nanocarriers	BALB/c mice	*E.C*	Increased bactericidal activity, no cytotoxicity, and promoted wound healing [49]
Ura56	Gold nanocarriers	Mice	*A.B, E.C., P. A*	Enhanced stability & bacterial-killing effects, good lytic activity [214]
Dpep	Silver nanocarriers	Mice	*E.C*	Interaction with bacterial membrane targeting LPS with 100-fold higher inhibition activity [215]

*A. B* Acinetobacter baumannii, *E.C* Escherichia coli, *P. A* Pseudomonas aeruginosa, *S. typhi* Salmonella enterica serovar Typhi

Cyclic AMP analogs of polymyxin B in nanocarriers reduced bacterial loads in an MDR *P. aeruginosa* model due to enhanced drug penetration into the epithelial lining fluids, thereby prolonging survival rates, indicating their potential to improve treatment efficacy for multidrug-resistant Gram-negative infections [39]. Polysaccharide nanocomplexes incorporated with polymyxin B were associated with better clinical outcomes for acute lung infection with effective biocompatibility and local bioactivity, highlighting how electrostatic assembly stabilized charged peptides and optimized their efficacy and safety profiles, as compared to free polymyxin in-vivo [193].

Colistin in nanoliposomes with chitosan demonstrated higher encapsulation yields, controlled colistin release during oral administration, alongside gastrointestinal resistance and adverse events [201]. in-vivo biodistribution and radiolabelling studies showed enhanced local intestinal uptake with adequate systemic absorption, suggesting the oral route as an alternative, compared to parenteral colistin administration [201]. Microenvironment-responsive nanocarriers can surpass challenges associated with colistin’s poor enteral bioavailability while mitigating gut mucosal cytotoxicity [201].

LL-37 encapsulated nanocarriers have promoted wound closure in an animal model infected by *E. coli*, thereby reducing bacterial contamination, and enhancing anti-inflammatory activity with accelerated wound closure in fibroblast cells and keratinocytes [160]. LL-37-containing PLGA nanoparticles improved peptides’ stability and bioavailability, thereby improving wound closure by enhancing angiogenesis and epithelial regeneration, alongside immune-modulating properties [209]. Therefore, nanoparticles-mediated AMP delivery can modulate endogenous host-defense peptides into viable clinical therapeutics by favouring better wound healing [209].

CO₂-expanded nanofibers encasing LL-37 helped to retain its microbial bioactivity by generating controlled 3D architectures, thereby promoting tissue regeneration by enhancing vascularization, cellular infiltration, and neo tissue formation following subcutaneous implantation [205]. These nanofibers also shifted macrophage polarization toward pro-regenerative M2 phenotypes, indicating adequate host responses. These findings highlight that CO2-expanded nanofibers can be used as an alternative platform for functional and 3D tissue engineering applications, and localized delivery as wound dressings [205].

In the zebrafish model, self-assembled melittin (MelNP) nanocarriers exhibited reduced bacterial loads and enhanced survival, illustrating that nano-assembly retained potency with partial blunted systemic toxicity [216]. While inorganic nanocarriers, i.e., protein-capped mesoporous silica nanoparticles, displayed topical bactericidal activity by releasing an optimized antimicrobial lipopeptide (i.e., SOAP, l-6-C5), validating the concepts of gated silica and AMP hybrids within an in-vivo skin infection model [217]. Nano-AMPs can outperform by modifying their design, focusing on stimulus response, and controlled and sustained release features.

Gold nanoparticle-DNA aptamer (AuNP-Apt) conjugated with AMP (AuNP-Apt-HPA3PHis) in treating *V. vulnificus* infections has disrupted bacterial membranes, thereby significantly reducing intracellular bacterial loads with 100% survival rates in infected mice, highlighting its therapeutic potential as a novel alternative drug delivery approach against multidrug-resistant *V. vulnificus* [218]. AuNP-Apt have shown enhanced stability and intracellular delivery of A3-APO(His) AMPs into *Salmonella Typhimurium*-infected HeLa cells and mice, eliminating bacterial colonization with 100% survival outcomes, highlighting its potential as a novel therapeutic strategy for treating intracellular bacterial infections [212]. Gold-silver hybrid nanocarriers (HA-P/Au/Ag) demonstrated a reduced number of bacterial colonies and inflammation within the lung tissues, having 100% survival rates with limited cytotoxicity [174].

One-step process (reduction-decomposition-reduction) was used to form ultra-small Dpep–Ag bio-nano clusters assembled of homogeneous peptides [215]. These nano-formulations demonstrated synergistic effects by fusing the membrane-active properties of the AMP Dpep alongside the bactericidal effects of silver, surpassing peptides-capped and commercially available silver nanoparticles [215]. Cationic peptides corona and smaller sizes, i.e., 2 nm, favoured selective targeting of LPS, thereby efficiently disrupting Gram-negative bacterial membranes, achieving MICs 100-fold lower against *E. coli* [215]. In the murine model, Dpep–Ag nanoclusters reduced bacterial burdens and promoted wound healing, exemplifying that metal-based AMP nanohybrids can optimize nano–bio interactions to achieve clinically relevant microbial profiles [215].

Collectively, these preclinical studies have provided robust scientific evidence that nano-formulations can uncouple antimicrobial efficacy from systemic adverse events, fundamentally reshape the PK/PD of encapsulated AMPs [174, 203]. Moreover, these trials have highlighted the bactericidal benefits of Nano-AMPs [174, 203]. However, despite these promising therapeutic and clinical outcomes, various challenges, such as achieving optimal pharmacokinetics and pharmacodynamics, ensuring bioavailability, and ensuring biocompatibility, must be addressed to overcome these hurdles, aiming to translate these promising preclinical results into effective clinical therapies for managing and treating multidrug-resistant Gram-negative pathogens.

### Clinical development status of Nano-AMPs

Despite extensive preclinical studies, currently, there are no registered human clinical trials of nanocarriers encapsulating AMPs targeting Gram-negative pathogens. Several antimicrobial peptides without nanocarriers, such as PL-5 (Peceleganan) [219, 220], melimine and Mel4, p2TA (AB103, Reltecimod) [221], and Dusquetide (IMX942, SGX942) [219, 220, 222–224] have undergone different phases of human testing, but these did not involve nanocarriers. In contrast, nanocarrier-based antibiotics have demonstrated regulatory feasibility against Gram-negative infections [225–227]. These different preclinical and clinical trials are an important translational precedent, but Nano-AMPs as novel alternative approaches remain preclinical.

Different Nano-AMP approaches have demonstrated promising drug delivery systems for Gram-negative pathogens in preclinical tests and appear closest to first-in-human readiness. LL-37-loaded chitosan-coated liposomes have shown enhanced antimicrobial activity and stability in *E. coli* and wound infection models, with clear potential for topical use [228, 229]. Self-assembled nonapeptides such as F3FT and N3FT demonstrated anti-inflammatory and antibacterial activity by disrupting bacterial membranes and generating ROS, eliminating intracellular pathogens with better biocompatibility, thereby offering a novel, promising strategy against resistant pathogens [230].

### Barriers associated with clinical translation of Nano-AMPs

#### Immunogenicity

AMPs demonstrate dual antibacterial and immunomodulatory properties [231]. However, their amphipathic and cationic nature can activate several aspects of the immune system, including the complement cascade, Toll-like receptors, or Mast cells [231]. Whilst the use of nanocarriers may mask these effects of the naked AMPs, nanocarriers themselves may provoke immune responses. They can also trigger complement and lead to opsonization, as well as inducing protein-corona–mediated immune recognition, development of, for example, anti-PEG antibodies [232–234]. Peptides surface conjugated within carriers may have altered epitope presentation, thereby prolonging exposure, which may heighten adaptive immune recognition [232–234]. Together, these factors may explain why several Nano-AMPs remain preclinical despite demonstrating compelling laboratory efficacy against resistant Gram-negative pathogens.

#### Strategies to mitigate the host’s immune responses

Peptide engineering, including sequence optimization (D-amino acids, cyclization, and epitope minimization), can reduce immunogenicity by limiting T-cell activation and hemolytic activity. Sequence editing (including charge and hydrophobic re-balancing) can prioritize host defence immunomodulation over membrane lysis [234]. For nano-carriers, immuno-stealthing, zwitterionic and hydrophilic alternatives and PEGylation may suppress protein corona formation, and biomimetic cloaking can limit complement deposition, thereby evading immune clearance and prolonging circulation [235]. Smart encapsulation approaches can release peptides only at infected sites, by exploiting bacterial enzymes, acidic pH, and ROS to minimize systemic manifestations [235, 236]. For controlled dosing regimens, these complementary approaches can favour co-delivery of immunomodulatory agents [235, 236]. Therefore, future progress will depend on standardizing the immunotoxicity assays, covering complement activation, antibody responses and cytokine production, and predictive computational models can be adapted to anticipate nano-AMPs stability and immunogenicity [236].

#### Regulatory, economic, and manufacturing barriers

Clinical translation of Nano-AMPs is hampered by the non-availability of harmonized regulatory frameworks [10, 237]. Variability regarding drug release kinetics, stability, and potency is paramount, further complicating regulatory submissions [238–240]. Cationic nanocarriers often facilitate bacterial membrane interactions due to electrostatic interactions, which are associated with complement activation and hemolysis, highlighting safety concerns [238–241]. At present, data regarding nanoparticles degradation, immunogenicity, and chronic manifestations are scarce, thereby leading to cautious regulatory stances for developing additional bridging studies [238, 240, 241]. All these hurdles reflect the absence of standardized manufacturing assays for Nano-AMPs, which must be an urgent priority.

Moving from benchside to clinical transition highlights scale-up challenges, such as laboratory methods and smaller batch emulsification, which may yield particle heterogeneity and peptide loading capacity, which are tightly scrutinized by regulators [242, 243]. Certain approaches, such as sterilization and lyophilization can destabilize nanocarriers, thereby affecting the drug’s pharmacological properties [242, 243]. PEGylated lipids, functionalized polymers, and metallic nanomaterials can amplify production costs, exacerbate economic inefficiencies [242, 243]. Nano-AMPs are often more expensive to manufacture due to peptide synthesis costs, alongside nanocarrier formulations, and intensive quality control [243, 244]. At present, more than 100 nanomedicines are marketed globally, and few of them are in clinical trials; none are Nano-AMPs for Gram-negative infections [243, 244].

#### Scalability strategies

Whilst conventional antibiotics have benefited from highly sophisticated and automated synthetic processes, yielding lower costs and larger-scale production [245], free AMPs remain constrained by the higher yields of solid-phase peptide synthesis (SPPS) and recombinant production, along with intrinsic instability to proteolysis [245]. Nano-AMPs also need to comply with good manufacturing practices (GMPs) and have strict quality control, drug loading, and endotoxin levels [246, 247]. Additionally, they require lyophilization with cryoprotectants to ensure storage stability [246, 247]. The inability of several laboratory procedures to be effectively transferred to continuous manufacturing, which increases batch-to-batch variability, makes scale-up a bottleneck [235, 246, 247]. Despite their potential for large-scale reproducibility, continuous-flow microfluidics and extrusion techniques are more expensive due to their financial requirements and regulatory uncertainty [235, 246–248].

The economic viability will ultimately depend on demonstrating how novel Nano-AMPs predicted and optimized by using advanced machine learning approaches will have better therapeutic and clinical outcomes, such as selected and targeted delivery, reduced dosing frequency, with minimal resistance emergence against Gram-negative pathogens, which is a current global health concern ( 235, 248).

## Computational approaches for Nano-AMP optimization

Computational approaches have provided essential tools for the identification and optimization of Nano-AMPs for combating AMR against Gram-negative bacteria [249, 250]. In-silico methods, including molecular dynamics (MD) simulations, molecular docking, and artificial intelligence (AI)-driven predictive models, can propose optimal designs for Nano-AMPs with enhanced specificity, stability, and antimicrobial potency [249–252]. Moreover, machine learning algorithms trained on vast AMP datasets can predict novel peptide sequences with ideal physicochemical properties, such as amphiphilicity, net charge, and amino acid chain length and sequence, crucial for membrane penetration and bacterial eradication [249–253]. Additionally, computational toxicity prediction and pharmacokinetics models help to assess the absorption, distribution, metabolism, and excretion (ADME) properties of Nano-AMPs, accelerating the drug development pipelines [252, 253].

In recent years, different computational approaches have begun to reshape the discovery of AMPs, providing pragmatic design strategies that extend beyond empirical optimization and screening [252, 253]. Discriminative and Generative models, such as AMPScanner v2 [254], AMP-GAN [255], iAMP-2L [256, 257], and Deep-AmPEP30 [258] have been successfully validated in-vitro and, in a few cases, in-vivo, with novel candidates demonstrating antimicrobial activity against multidrug-resistant *E. coli*, *P. aeruginosa*, and *K. pneumoniae*, respectively.

Combining machine learning with nanobiotechnology offers practical translational pathways, aligning novel peptide discovery with viable drug delivery strategies. Integrating AI-predicted AMPs into various nanocarrier systems (such as liposomes, PLGA nanoparticles, solid lipid nanoparticles, hybrid gels, and peptide self-assemblies) may enhance their stability, enable targeted delivery, and reduce systemic toxicity [62–65]. Recently, AI has closed the loop from in-silico designs to in-vivo validation for AMP-encapsulated nanocarriers [259]. AI-integrated “nanoarchitectonics” frameworks can be used to couple target profiling, machine learning guided nanocarrier surface engineering, pharmacokinetic and pharmacodynamics simulation for tuning nanocarrier optimal size, surface charge, PEG density, microbial membrane interactions, and can facilitate AMP loading, aggregation, and hemolysis [259, 260].

For characterization, AI can make the nanocarrier behaviour for AMPs predictable and quantifiable [261]. Automated formulation platforms can help to generate in-vitro and in-vivo datasets for the nanocarrier’s uptake and efficacy [261]. AI-assisted models, such as PBPK and PINN, can infer kinetic parameters, e.g., permeability, tissue retention, thereby facilitating dosing and delivery predictions without in-vivo calibrations [262]. Moreover, AI-based protein-corona analytics can help to connect corona composition for immune recognition, engineering AMP nanocarriers for reduced opsonization, and improved local tissue accumulation [263, 264]. Altogether, advanced AI approaches can help to optimize biodistribution and endosomal escape into designable nanocarrier properties, thereby de-risking clinical translation [265].

However, computational methods for designing Nano-AMPs have several drawbacks that need to be resolved for successful clinical translation, notwithstanding their benefits [249–253]. The reliability of AI-driven predictions is a significant problem because existing training datasets frequently lack diversity for elucidating host–pathogen interactions that affect AMP efficacy in-vivo [250–253]. There is a need for experimental validation because unmodeled elements like protein binding, immunological reaction, and nanoparticle aggregation might cause differences between in-silico predictions and actual antimicrobial activity [249–253].

Despite these advances, not a single machine learning-based pipeline has advanced Nano-AMPs to registered human trials, making this modality a frontier for future research work. Most machine learning platforms, such as UniAMP [266], GAN-for-AMP-Design [267], AMPlify [268, 269], and AMPsphere [270], have only been validated at the peptide synthesis and antimicrobial assay levels, without systematic integration of nanocarriers. Although the reported examples of different AMP-nanocarriers are promising, they remain at preliminary stages, thereby underscoring challenges in terms of scalability, manufacturing, and regulatory classification [271–273]. At present, not a single machine learning based AMP pipeline has yet advanced different Nano-AMPs to registered human trials, making this modality a frontier for future research work.

To accelerate clinical translation, future developments ought to concentrate on combining machine learning with molecular simulations, multi-scale modelling techniques, and experimental feedback loops to improve prediction accuracy [274–277]. The creation of high-throughput screening platforms that integrate automated synthesis, testing, and computational design will also be essential in closing the gap between in-silico predictions and clinical implementations against Gram-negative bacteria that are resistant to conventional drugs by overcoming these obstacles [274–277].

## Current gaps and future directives

Nano-AMPs can offer modular and highly potent alternatives to AMPs or traditional antibiotics. However, various systemic bottlenecks have continued to impede their clinical advancements. Despite the rapid growth of preclinical data, a significant translational gap remains. Most studies continue to focus on demonstrating potency across various in-vitro and in-vivo models, yet no human trials of Nano-AMPs have been initiated to date [245]. This gap reflects unresolved and critical challenges, including non-selectivity, susceptibility to proteolysis, narrow therapeutic windows, and long-term safety concerns, thereby limiting their regulatory momentum [278–280].

Immunological concerns associated with Nano-AMPs are not fully understood yet. AMPs and the nanocarriers, i.e., polymers, lipids, or metal-based, may activate either innate and adaptive immune responses, thereby accelerating their rapid immunological clearance, especially in response to repeated dosing [278–281]. Certain strategies, such as identification, optimization, and AMPs’ sequential humanization using different advanced machine learning approaches, and PEGylation, are being explored. However, there are no universal and systematic immunoprofiling protocols [237, 260, 278–280]. Collectively, these issues explain why Nano-AMPs remain in the preclinical stage, despite the urgent need for new therapies targeting multidrug-resistant infections [237, 281].

To bridge translational barriers, future trials must prioritize harmonized assay frameworks, immunologically informed designs, and scalable manufacturing strategies [279, 280, 282]. Integrating advanced machine learning and deep generative models alongside peptide engineering for accelerating AMP discovery with ideal biochemical and carrier compatibilities [237, 260, 277]. Microenvironment-triggered signals, such as enzymes, pH, and ROS, must be harnessed for the selective targeting to localize AMP activity at infection sites and minimize systemic exposure [282]. Employ advanced stealth or functionalization nanocarrier strategies to limit long-term immunogenicity and cytokine profiling, and conduct early preclinical screening for complement activation, immune memory, and anti-drug antibodies [282].

Standardization of in-vitro and in-vivo potency assays is essential, including controls for particle charge, size, and drug-release kinetics. Adoption of advanced microfluidic platforms and manufacturing technologies will reduce cost, which supports scalability and reproducibility, thereby bridging wet-lab innovations and clinical outcomes [282, 283]. Early-phase clinical trials should focus on localized delivery for Gram-negative infections, using biomarker endpoints and microbial burdens, alongside safety metrics to limit systemic effects [282, 283]. By addressing manufacturability and minimizing non-specific immune responses, Nano-AMPs can establish a credible path toward first-in-human trials with the potential for effective clinical outcomes against resistant Gram-negative pathogens [248].

## Conclusion

Nano-AMPs have demonstrated significant potential as alternative therapeutic strategies for combating antimicrobial resistance, exhibiting potent bactericidal activity through mechanisms like disruption of microbial membranes and intracellular targeting. However, challenges such as production scalability, stability, rapid degradation, and host cytotoxicity must be addressed to highlight their therapeutic applications. Addressing these concerns requires a combination of advanced machine learning approaches, regulatory innovations, and interdisciplinary research to ensure the successful clinical translation of Nano-AMPs carriers from bench to bedside.

Nano drug delivery systems can be used to enhance AMPs’ efficacy, improving AMP bioavailability and biocompatibility, limiting cytotoxic events, and enabling selected and targeted delivery to Gram-negative pathogens. Nano-AMPs are specifically suited to overcome various intrinsic and extrinsic resistance barriers by enhancing AMP stability, penetration, and antimicrobial potency.

The synergistic effects of Nano-AMPs, along with AMPs' intrinsic bactericidal properties in combination with nanobiotechnology's precision delivery and advanced machine learning approaches, can make them novel therapeutic approaches over conventional antibiotics and free AMPs. By addressing the limitations of traditional antimicrobial therapies and mitigating resistance development, Nano-AMPs offer a viable and broad-spectrum solution for combating infections caused by multidrug-resistant Gram-negative pathogens.

## Data Availability

No new data were generated or analysed in this study. All data supporting the findings of this review are available within the article and its cited references.

## Abbreviations

*A. B*: *Acinetobacter baumannii*
AI: Artificial Intelligence
AMPs: Antimicrobial peptides
AMR: Antimicrobial resistance
AgNPs: Silver nanoparticles
AuNPs: Gold nanoparticles
AuNP-Apt-HPA3Phis: Gold nanoparticle-DNA aptamer
CD: Cyclodextrin
DNA: Deoxyribonucleic acid
*E.C.*: *Escherichia coli*
E. faecium: *Enterococcus faecium*
EPS: Exopolysaccharide
ESBLs: Extended-spectrum β-lactamases
GMPs: Good Manufacturing Practices
HGT: Horizontal gene transfer
K. P: *Klebsiella pneumoniae*
LPS: Lipopolysaccharides
MelNP: Self-assembled melittin nanocarriers
MD: Molecular dynamics
MDR: Multidrug-resistant
NLCs: Nanostructured lipid carriers
*P. A*: *Pseudomonas aeruginosa*
PDHs: Peptide-dendrimer hybrids
PEG: Poly (ethylene glycol) (PEG)
PSME3: Proteasome activator subunit 3
PLA: Poly (lactic acid)
PLGA: Poly (lactic-co-glycolic acid)
RES: Reticuloendothelial system
RNA: Ribonucleic acid
ROS: Reactive oxygen species
SLNs: Solid lipid nanoparticles
*S. typhi*: Salmonella enterica serovar Typhi
*V. vulnificus*: *Vibrio vulnificus*
*V.C*: *Vibrio cholerae*
WHO: World Health Organization
